# Ten-gene signature reveals the significance of clinical prognosis and immuno-correlation of osteosarcoma and study on novel skeleton inhibitors regarding MMP9

**DOI:** 10.1186/s12935-021-02041-4

**Published:** 2021-07-14

**Authors:** Weihang Li, Ziyi Ding, Dong Wang, Chengfei Li, Yikai Pan, Yingjing Zhao, Hongzhe Zhao, Tianxing Lu, Rui Xu, Shilei Zhang, Bin Yuan, Yunlong Zhao, Yanjiang Yin, Yuan Gao, Jing Li, Ming Yan

**Affiliations:** 1 Department of Orthopaedics, Xijing Hospital, The Fourth Military Medical University, Xi’an, People’s Republic of China; 2 School of Aerospace Medicine, Fourth Military Medical University, 169 Chang Le Xi Road, Xi’an, 710032 Shaanxi China; 3 Department of Intensive Care Unit, Nanjing First Hospital, Nanjing Medical University, Nanjing, 210006 Jiangsu China; 4 State Key Laboratory of Oncology in South China, Collaborative Innovation Center for Cancer Medicine, Sun Yat-Sen University Cancer Center, Guangzhou, 510060 People’s Republic of China; 5 Hou Zonglian Medical Experimental Class, Xi’an Jiaotong University, Xi’an, Shaanxi China; 6 Department of Endocrinology, Shanghai National Research Center for Endocrine and Metabolic Disease, State Key Laboratory of Medical Genomics, Shanghai Institute for Endocrine and Metabolic Disease, Ruijin Hospital. Shanghai Jiaotong University School of Medicine, Shanghai, People’s Republic of China; 7 Department of Spine Surgery, Daxing Hospital, Xi’an, Shaanxi China; 8 College of Clinical Medicine, Jilin University, Changchun, China; 9 Department of Hepatobiliary Surgery, National Cancer Center/National Clinical Research Center for Cancer/Cancer Hospital, Chinese Academy of Medical Sciences and Peking Union Medical College, Beijing, 100021 China

**Keywords:** Biomarkers, Differential gene expression analysis, Inhibitor, Matrix metalloproteinase-9, Virtual Screening

## Abstract

**Objectives:**

This study aimed to identify novel targets in the carcinogenesis, therapy and prognosis of osteosarcoma from genomic level, together with screening ideal lead compounds with potential inhibition regarding MMP-9.

**Methods:**

Gene expression profiles from GSE12865, GSE14359, GSE33382, GSE36001 and GSE99671 were obtained respectively from GEO database. Differentially expressed genes were identified, and functional enrichment analysis, such as GO, KEGG, GSEA, PPI were performed to make a comprehensive understanding of the hub genes. Next, a series of high-precision computational techniques were conducted to screen potential lead compounds targeting MMP9, including virtual screening, ADME, toxicity prediction, and accurate docking analysis.

**Results:**

10 genes, MMP9, CD74, SPP1, CXCL12, TYROBP, FCER1G, HCLS1, ARHGDIB, LAPTM5 and IGF1R were identified as hub genes in the initiation of osteosarcoma. Machine learning, multivariate Cox analysis, ssGSEA and survival analysis demonstrated that these genes had values in prognosis, immune-correlation and targeted treatment. Tow novel compounds, ZINC000072131515 and ZINC000004228235, were screened as potential inhibitor regarding MMP9, and they could bind to MMP9 with favorable interaction energy and high binding affinity. Meanwhile, they were precited to be efficient and safe drugs with low-ames mutagenicity, none weight evidence of carcinogenicity, as well as non-toxic with liver.

**Conclusions:**

This study revealed the significance of 10-gene signature in the development of osteosarcoma. Besides, drug candidates identified in this study provided a solid basis on MMP9 inhibitors’ development.

**Supplementary Information:**

The online version contains supplementary material available at 10.1186/s12935-021-02041-4.

## Introduction

Osteosarcoma (OS), one of the most common malignant neoplasm, accounts for 20–40% of all bone cancers, which is characterized by the direct formation of osteoid tissue, osteoid- and spindle-shaped matrix cell in immature bones [1]. The main clinical manifestation of OS patients frequently suffers from swelling and bone pain, while systematic symptoms, such as weight loss, pallor, night sweats, fever and anorexia are seldomly seen [2]. Osteosarcoma appears mainly in childhood and adolescents, together with an overall incidence of 0.3–0.4/100000 individuals per year [3, 4].

Currently, the main clinical treatment for patients with OS included surgery, radiotherapy, and chemotherapy. Since the 1970s, with the introduction and invention of neoadjuvant chemotherapy and limb salvage surgery for OS, the overall survival rate had improved significantly. Despite advances in surgical technique and targeted chemotherapy, optimal treatment outcomes in OS are still negatively impacted by infection, tumor immunity, complications [5–8]. The overall prognosis of patients with OS is only 20%, especially in patients with metastasis disease or tumor recurrence [9]. Overall expression as well as imbalance of MMP9 are associated with a variety of diseases, regulating and inhibiting MMP9 is an essential therapeutic approach to treat various diseases including cancer. Therefore, MMP9 inhibitors could be regarded as anticancer drugs [10]. JNJ0966, GS-5745, two chemotherapy drugs, were selective inhibitors regarding matrix metalloproteinase-9 (MMP-9), had shown prospective view in treatment of encephalomyelitis, ulcerative colitis and gastric cancer [11, 12]. However, only few inhibitors had a relatively promising function in inhibiting MMP9. Meanwhile, researches based on MMP9 inhibitors regarding osteosarcoma had hardly been reported before, which could make significance in targeted chemotherapy regarding osteosarcoma. Therefore, based on the fact that poor prognosis and lack of effective targeted therapies as well as tumor-related biomarkers in OS, there is an urgent need to explore novel predictive and prognostic biomarkers as well as discover lead compound inhibitors regarding OS, in order to make a comprehensive understanding of oncogene and eventually improve the prognosis of patients.

Recent decades, with the rapid development of high-throughput technology, large-scale RNA sequence transcriptome data as well as gene microarray chips, bioinformatics analysis have displayed a promising view to identify prognostic genes, elucidate the oncogenic mechanism for plenty of neoplasms and finally improve the treatment of cancers [13, 14]. It helps us study the initiation, progression and metastasis of different neoplasms under transcriptome level, which makes it possible to explore the molecular mechanisms and discover disease-specific biomarkers of OS in this study. Natural compounds and their derivatives play an essential role in today’s pharmacologic market [15, 16]. Due to their malleable, convertibility and readily available property, they have made a great contribution to medication design and improvement in treating cancers [17–19]. To the best knowledge by consulting amount of literatures, current researches of OS using bioinformatics mainly focused on studying the hub genes among metastasis and non-metastasis osteosarcoma, that is to say, current research samples were mostly osteosarcoma samples whereas normal bone tissues were not included in study, while another extremely vital research about hub genes among osteosarcoma and normal bone tissue, had hardly been reported. Consequently, the combination of bioinformatics and structural biology study were performed in this study to accelerate the understanding of hub genes and discovery of inhibitors regarding OS.

**Fig. 1:**
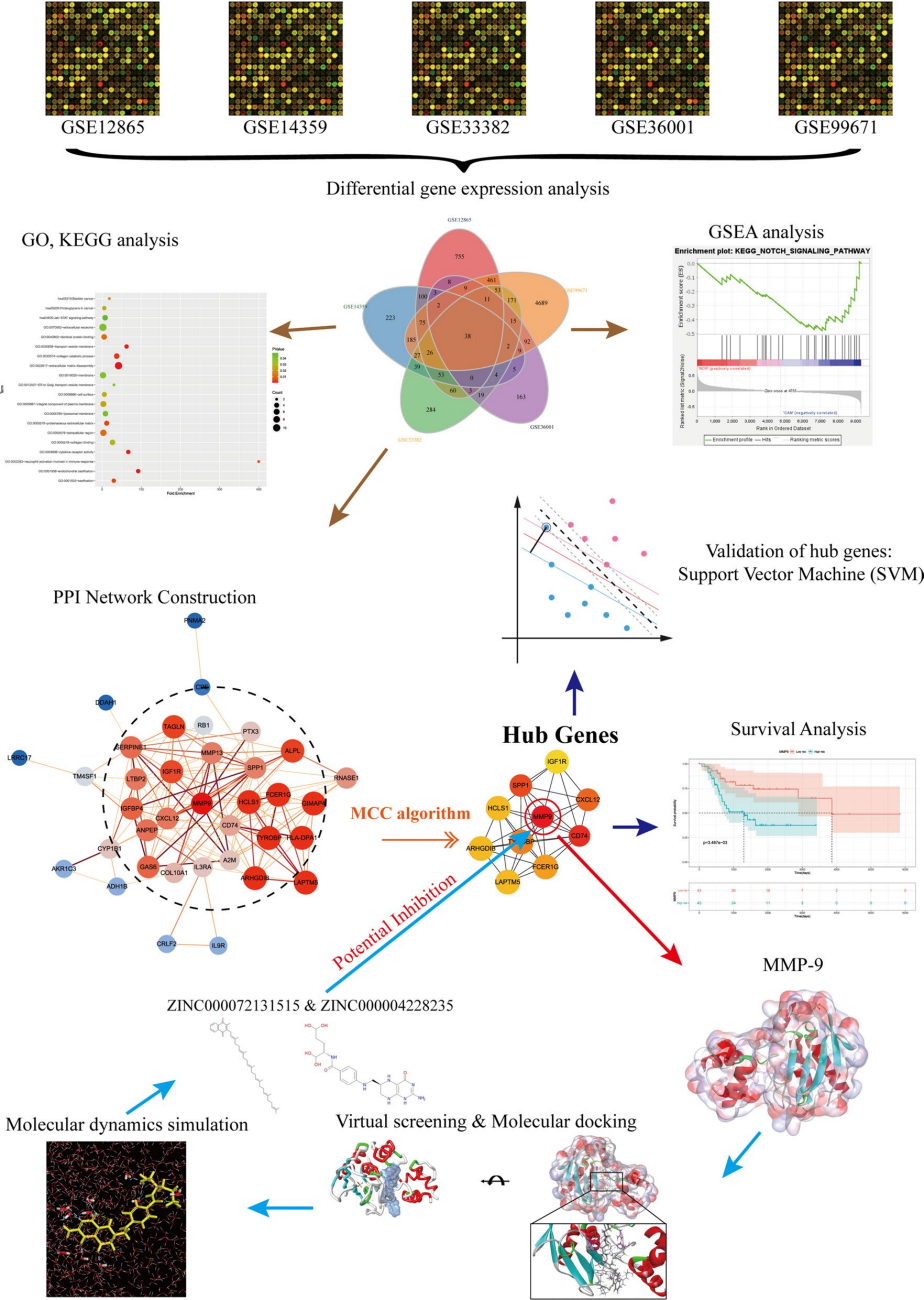
The whole diagram and workflow of this study. MMP9: Matrix metalloproteinase-9; GO: Gene Otology; KEGG: Kyoto encyclopedia genes and genomes; GSEA: Gene set enrichment analysis; PPI: protein–protein interaction; SVM: Support vector machine

The accurate differential diagnosis is the key to conduct precise prognosis as well as target-therapy [20]. This study firstly aimed to identify hub genes using differential expression method in the occurrence of osteosarcoma. 5 mRNA microarray datasets (GSE12865, GSE14359, GSE33382, GSE36001, GSE99671) involving osteosarcoma and correspondent normal bone samples were downloaded from Gene Expression Omnibus (GEO). Next, the mutual differentially expressed genes (DEGs) were screened with Venn analysis. Gene Ontology (GO), Kyoto Encyclopedia of Genes and Genomes (KEGG) methods were performed to discover molecular function changes and abnormal signaling pathways by displaying their molecular function, biological process and cellular component. Protein–protein interaction (PPI) analysis was conducted further to find the most-linked genes with OS. Subsequently, this study further performed machine learning and survival analysis in third party TCGA and GEPIA2 database to validate and confirm our results. Finally, this study conducted structural biology analysis including a series of structural and chemical methods to screen ideal lead compounds based on identified hub genes, which may have biological effects in the treatment of OS and provide new ideas for medication development in pharmacologic market. This study provided a set of method flow to understand the cause and molecular events of OS, then offered a list of drug candidates with pharmacological properties from ZINC15 database, which made a solid basis theory for gene products inhibitors’ research. The whole diagram and workflow of this study was shown in Fig. 1.

## Materials and methods

### Datasets from GEO and TCGA database

Gene expression profiles of GSE12865, GSE14359, GSE33382, GSE36001 and GSE99671 were downloaded from Gene Expression Omnibus (https://www.ncbi.nlm.nih.gov/geo/). Multiple sample sets were used in this study to avoid clinical and race bias among different studies. The inclusion criteria were as follows: samples which were normal bone tissues or osteosarcoma tissues with non-metastasis were included for analysis. Altogether, this study included 2 normal samples and 12 osteosarcoma samples in GSE12865; 2 normal samples and 10 osteosarcoma samples in GSE14359; 3 normal samples and 84 osteosarcoma samples in GSE33382; 6 normal samples and 8 osteosarcoma samples in GSE36001; 18 normal samples and 18 osteosarcoma samples in GSE99671. As for TCGA dataset (https://portal.gdc.cancer.gov/), all data in osteosarcoma and corresponding clinical information were downloaded by “TCGAbiolinks” package in R [21]. It had 379 recorded clinical samples and totally 88 samples with gene expression profile, which was annotated to a reference transcript set of Human hg38 gene standard track.

### Gene expression profiles’ preprocess

This study used raw data (“.CEL” file format: GSE12865, GSE14359) and “series_matrix_file” (“.txt” file format: GSE33382, GSE36001, GSE99671) from these 5 GSE datasets. The method of quality control was performed by R (“affy”, “affyPLM” package), we evaluated the samples quality of GSE12865 and GSE14359 datasets through calculating their normalized unscaled standard errors (NUSE). After that, we conducted robust multi-array average (RMA) algorithm using “rma” function in R to perform background correction and normalization of gene expression profile. Probes in these 5 GSE datasets and TCGA database were converted into corresponding gene symbols based on manufacture-provided annotation files, probe sets without corresponding genes were removed, as for different probes targeting the same gene, the highest expression value of all its corresponding probes were retained. Lastly, “batch effects” generated in these 5 GSE datasets were detected and removed using “combat” function in R (“sva”, “pamr” package).

### Identification of DEGs

The differentially expressed genes between osteosarcoma and normal samples were screened using R (“limma” package). “limma” package is one of the most widely used packages in Bioconductor repository to identify DEGs, which allows researchers to compare two or more datasets in GEO in order to discover DEGs through experimental conditions. The adjusted P-values and false discovery rate (Benjamini–Hochberg algorithm) were applied to provide a balance guideline between discovery of statistically significant genes and limitations of false-positives. Each time we compared two groups to identify DEGs between osteosarcoma samples and normal samples in each GSE datasets. The DEGs were calculated and screened by filtering adjusted P-value < 0.05 and |logFC| (fold change) > 1, which was considered as statistically significant definition. Subsequently, Venn plot analysis among DEGs in these 5 datasets was conducted further to obtain a more precise result (“VennDiagram” package in R).

### GO and KEGG analysis on DEGs

Functional annotation and interpretation were performed in DAVID database (Database for visualization, annotation and integrated discovery, http://david.abcc.ncifcrf.gov/) to discover biological meaning of genes [22]. Gene Ontology (GO) is a powerful method to analyze biological process, cellular component and molecular function among different genes. Kyoto Encyclopedia of Genes and Genomes (KEGG), a basis for gene function and genomic information links, is used to analyze information about signaling pathway relationships. GO and KEGG were performed for identified DEGs in DAVID database, P < 0.05 was set as threshold for statistically significant definition.

### Gene set enrichment analysis (GSEA)

Gene Set Enrichment Analysis (GSEA, http://software.broadinstitute.org/gsea/index.jsp [23]) was conducted from overall genes to further determine other essential biological functions or signaling pathways which may be ignored by differential analysis, the annotated gene set c2.cp.kegg.v7.1. symbol was selected as a reference. Gene size ≥ 20, P < 0.05, and |enrichment score (ES)|> 0.40 were set as the cutoff criteria.

### Construction of PPI network and screening of hub genes

This study used STRING database (Search Tool for the Retrieval of Interacting Genes, https://string-db.org/ [24]), which is an online repository designed for predicting protein–protein interactions (PPI), to construct a PPI network of total DEGs. Then, Cytoscape software (version 3.8.0, an open source bioinformatics software platform for visualizing interaction networks) was performed to load the PPI network generated in STRING database. “Cytohubba” and “MCODE” plug-in in Cytoscape was conducted to screen hub genes and modules among total DEGs, respectively. Within a co-expression network, Maximal Clique Centrality (MCC) algorithm in “cytohubba” plug-in was reported to be the most effective method in finding hub genes, which aims to identify key targets and sub-network in a complexity network [25]. Finally, the genes with top 10 MCC values were considered as hub genes.

### Establishment of SVM to validate the reliability of hub genes

In order to confirm the predictive reliability of these hub genes, the dataset GSE33382 was set as training set to make a Support Vector Machine (SVM) classifier. 10 hub genes were used to train the SVM classifier, “e1071” package in R was conducted for training. To test the stability and transferability of trained SVM, the generated SVM classifier was verified by using independent testing datasets: GSE12865, GSE14359, GSE36001 and GSE99671. The classification effect of SVM was determined according to sensitivity (Se), specificity (Sp), receiver operating characteristic curve (ROC) and area under curve (auc) (“pROC” package in R). Five-fold cross validation was utilized when machine learning in order to obtain the most fitting equation as well as the most accurate results of the testing set.

### Gene signature robustness verification

To validate the 10-gene signature robustness, the “RiskScore” was calculated for each sample based on the gene expression levels in TCGA database through weighting Cox regression coefficients. The “timeROC” package in R was performed to depict the ROC of the “RiskScore” for prognostic classification. Patients were categorized as low-risk and high-risk group according median value of RiskScore. Survival analysis between two risk groups was conducted with “survival” package in R.

### Gene signature model evaluation

To further assess the relationship between the RiskScore of the gene signature model and the 29 immune gene set level, the single-sample gene-set enrichment analysis (ssGSEA) [26] (“estimate”, “GSVA” package in R) was carried to calculate the immune scores (immune cell infiltration level), stromal scores (stromal content), estimate scores as well as tumor purity for each sample. Simultaneously, expression of HLA-genes and immune check point genes were compared between high- and low-risk groups by ANOVA test to figure out the relationship between HLA genes, immune check point genes and different risk groups.

### Evaluation of prognostic values of hub genes in third-party database

Kaplan–Meier univariate survival analysis was performed in R (“survival”, “survminer” package) to explore the relationship between overall survival and hub genes in patients with osteosarcoma in TCGA database. In this study, only patients with completed follow-up times were selected for survival analysis and patients were divided into two groups (group-high and group-low) based on the median expression value of hub genes. Moreover, the association between disease-free survival (DFS) and hub genes was analyzed using the online tool GEPIA2 (http://gepia.cancer-pku.cn/) [27]. The survival-related hub genes with log-rank P < 0.05 were regarded as statistically significant.

### Prediction of overall survival in clinical application

The independent prognostic ability of the gene signature was evaluated by univariate and multivariate Cox analysis. An innovative nomogram plot was depicted based on the result of multivariate Cox analysis (“rms” package in R). Next, calibration plots of observed vs. predicted probabilities of 3-, 5-, 10- year’s overall survival of osteosarcoma patients was conducted to determine the accuracy of the predictive ability of these gene signature. The concordance index (C-index) was used to depict the discrimination of the model.

### Validation of MMP9 gene expression patterns in different cancers

In order to confirm the value of MMP9 identified in this study as well as provide a solid practical and theoretical basis for the subsequent screening of targeted inhibitors. This study analyzed the MMP9 expression value in different cancers, including SARC (sarcoma), GBM (glioblastoma multiforme), KIRC (kidney renal clear cell carcinoma), LUSC (lung squamous cell carcinoma), COAD (colon adenocarcinoma) and BLCA (bladder urothelial carcinoma). The expression level of MMP9 between normal and tumor samples in each cancer were plotted as box plot graph. P-value cutoff was set as 0.01 and jitter size was set as 0.4.

### Docking software and ligand repository

This study used Discovery Studio software (version 4.5) for structural biology research, which is a suite of software for simulating small molecules and macromolecules system. It is a new generation of molecular modeling and environmental simulation software for the life science field [17]. Discovery Studio (DS) is developed as a reliable software aiming to provide protein modeling, optimization and medication design tools by applying structural chemical and structural biology computation. Large amount of lead compounds as well as drug candidates were screened through this method. Natural lead compounds used in this study were arose from ZINC15 database, which was a powerful natural product repository for screening, development and research of ligands. It was also a free database of commercially available compounds offered by the Irwin and Shoichet Laboratories among department of Pharmaceutical Chemistry, University of California, San Francisco (San Francisco, California, USA).

### Structurally virtual screening using libdock

The 1.80 Å crystal structure of human MMP9 (PDB ID: 5UE4 [12]) was downloaded from RCSB Protein Data Bank and then imported into systematic working circumstance of DS. The crystal structure of MMP9 was shown in Additional file 1: Figure S1. Ligand-binding pocket region of MMP9 was selected as the binding site to screen compounds which could potentially inhibit MMP9. Virtual screening was carried out through libdock module in DS4.5. Libdock is a rigid-based docking program, which calculates hotspots for the protein using a grid placed into the binding site with polar and apolar probes (San Diego, CA, USA). Next, the hotspots are further employed to align the ligands to form favorable interactions. The Smart Minister algorithm and CHARMm forcefield (Cambridge, MA, USA) were performed for ligands minimization [28]. After that, all the ligand postures were ranked based on ligands score. The MMP9 protein was prepared for docking program by removing crystal water and other hetero atoms around it, followed by addition of hydrogen, ionization, protonation and energy minimization. The binding site of prepared protein MMP9 was defined from “edit binding site” option on the receptor-ligand interaction tool bar. Through the initial ligand docked with MMP9, the active binding site for docking could be generated. Virtual screening was then carried out by docking all the prepared ligands from ZINC15 repository at the defined active binding site. All the docked postures were ranked and grouped by compounds’ name based on libdock score.

### ADME and toxicity predictions

ADME (absorption, distribution, metabolism and excretion) module in DS4.5 was conducted to calculate compounds’ ADME pharmacological properties, including aqueous solubility, blood–brain barrier penetration, human intestinal absorption, cytochrome P4502D6 (CYP2D6) inhibition, hepatotoxicity and plasma protein binding level. TOPKAT (Toxicity Prediction by Komputer Assisted Technology) module of DS4.5 was also carried out to calculate the toxicity as well as predict other properties of these compounds, such as Ames mutagenicity, FDA carcinogenicity level (male rat, female rat, male mouse, female mouse), and weight of evidence carcinogenicity. These pharmaceutical properties of compounds were comprehensively assessed when selecting drug candidates for MMP9.

### Highly precise docking and visualization

CDOCKER module in DS4.5 was employed for molecular docking study. CDOCKER is a highly precise docking strategy for ligands and proteins based on CHARMm forcefield, which could calculate and provide accurate energy results for analysis. Receptor was held rigid while ligands were allowed to be flexible during the docking process. For each complex posture, the interaction energy in CHARMm forcefield, which indicated ligand binding affinity, was calculated. The crystal structure of MMP9 obtained from PDB was prepared by removing water molecules during rigid and semi-flexible docking process in case that the fixed molecule water might affect the formation of receptor-ligand complex [29]. To testify the reliability of docking system used in this study, the initial ligand in MMP9 was extracted from the binding site and then re-docked into MMP9 through CDOCKER process, root mean squared deviation (RMSD) between these two conformations was calculated to prove the reliability. The active binding site sphere of MMP9 was defined as the regions which came within 5 Å radius from the geometric centroid of the ligand JNJ0966 in MMP9 (PDB ID: 5UE4). The candidate ligands from ZINC15 repository were docked into the binding site of MMP9. The docking poses value was set as 10 and pose cluster radius was set as 0.5 in order to ensure that the ligands conformation was as diverse as possible. Different postures of each MMP9-ligand complex were generated, analyzed and visualized on the basis of CDOCKER interaction energy. The clusters ranking was performed based on the lowest energy representative for each cluster as well as the most appropriate orientation. According to visualization and calculation, ligands with the lowest energy together with appropriate posture were selected for further investigation.

### Cell lines and reagents

Human osteosarcoma cell lines (HOS: CL-0360 and MG-63: CL-0157) were obtained from Procell Life Science & Technology Co., Ltd. These cell lines were cultured in high-glucose DMEM (Procell, Cat.no.), containing 10% fetal bovine serum (FBS, Gemini, USA) and 100 units/mL penicillin and 100 mg/mL streptomycin, under normal cell culture conditions (37 ℃ and 5% CO2). ZINC000072131515 was provided by Selleck Chemical Co. (Cat.no. S5082). ZINC000072131515 was dissolved in DMSO to obtain the stock solution, then appropriate culture medium was respectively added into the stock solution to configure different concentrations of ZINC000072131515 cell culture medium.

### In vitro scratch assay

HOS and MG-63 were seeded and cultured in 6-well plate, when the degree of fusion reached 90%, a 1 mL pipette tip was used to make a consistent cell-free area. Then PBS was used to rinse the cell debris, and serum-free medium were changed to culture and 10 μmol/L of ZINC000072131515 were used to treat the cells at 0, 12, 24 h. After corresponding time, we captured images of the scraped area with phase contrast microscopy and measured the wounds and scratch width.

### CCK-8 assay

The cells viability of human osteosarcoma cell lines (HOS and MG-63) was assessed by Cell Counting Kit-8 (CCK-8) (ApexBio, USA). Cell lines were plated into 96-well culture plates with a density of 5000 cells/well for overnight, then different doses of ZINC000072131515 were treated with cells for 48 h. The concentration gradients of each treatment were 0 μmol/L, 1 μmol/L, 2 μmol/L, 4 μmol/L, 8 μmol/L, 16 μmol/L, 32 μmol/L, 64 μmol/L, respectively. Cells were cultured for 3 h after addition of 10 μL/well CCK-8, and then wavelength of 450 nm was applied to measure the OD value of each well on the microplate reader (BioTek instrument, Synergy H1, USA).

### Colony formation assay

HOS and MG-63 cell lines were inoculated into 6-well culture plate with the density of 600 cells per well. After 24 h in culture, the concentration of DMSO was configured with 10 μmol/L and 20 μmol/L, respectively, and concentration of DMSO was less than 0.1%. Based on this dilution ratio, the influence of DMSO on cells could be neglected. After 2 weeks of cultivation, the formed colonies were rinsed with phosphate-buffered saline and fixed in 4% paraformaldehyde, then 0.5% crystal violet solution was used to stain the developed colonies for half an hour. Lastly, microscopic examination was used to count colony with more than 50 cells.

## Results

### Gene expression profiles’ detection

Normalized unscaled standard errors (NUSE) of GSE12865 and GSE14359 were calculated and plotted for each sample in Additional file 2: Figure S2, results illustrated that the raw data used in this study was highly reliable in chip quality. After background correction, normalization, and gene symbols correspondence, boxplot or violin plot of these 5 GSE series were displayed to detect the expression value of these data. Results showed that the median expression value of each dataset were on a straight line, and gene expression value accorded with normal distribution, indicating that the data processed in this study could be analyzed for further investigation.

### Elimination of batch effects and principle component analysis in GSE series

Microarray experiments were expensive and time-consuming, lots of researches used multi arrays with experiments at different time, different array chargers or even different microarray platforms, causing some problems when researcher analyzed multiple datasets. Non-biological experimental variation or “batch effects” were commonly observed among multiple batches of microarray experiments [30]. Firstly, principle component analysis (PCA) was carried out in this study to reduce dimension of these 5 GSE series in order to observe whether batch effects existed among them (Additional file 3: Figure S3A). Results illustrated that they clustered separately, therefore, there was a significant biological difference among those 5 datasets. Then, this study performed “combat” function (“sva” and “pamr” package in R) to eliminate batch effects generated in these series. Next, PCA was performed again to validate the processed results (Additional file 3: Figure S3B), scatter diagram displayed non-difference in point distribution on image, they clustered together with each other. Meanwhile, QQ plot and density plot generated by eliminating batch effects were visualized in Additional file 3: Figure S3C. Subsequently, we made PCA on the processed matrix to observe the difference between different phenotypes (normal versus tumor), and results illustrated that normal samples could be distinguished from tumor samples on PCA1 axis (Additional file 3: Figure S3D).

### Identification of DEGs in OS

**Fig. 2:**
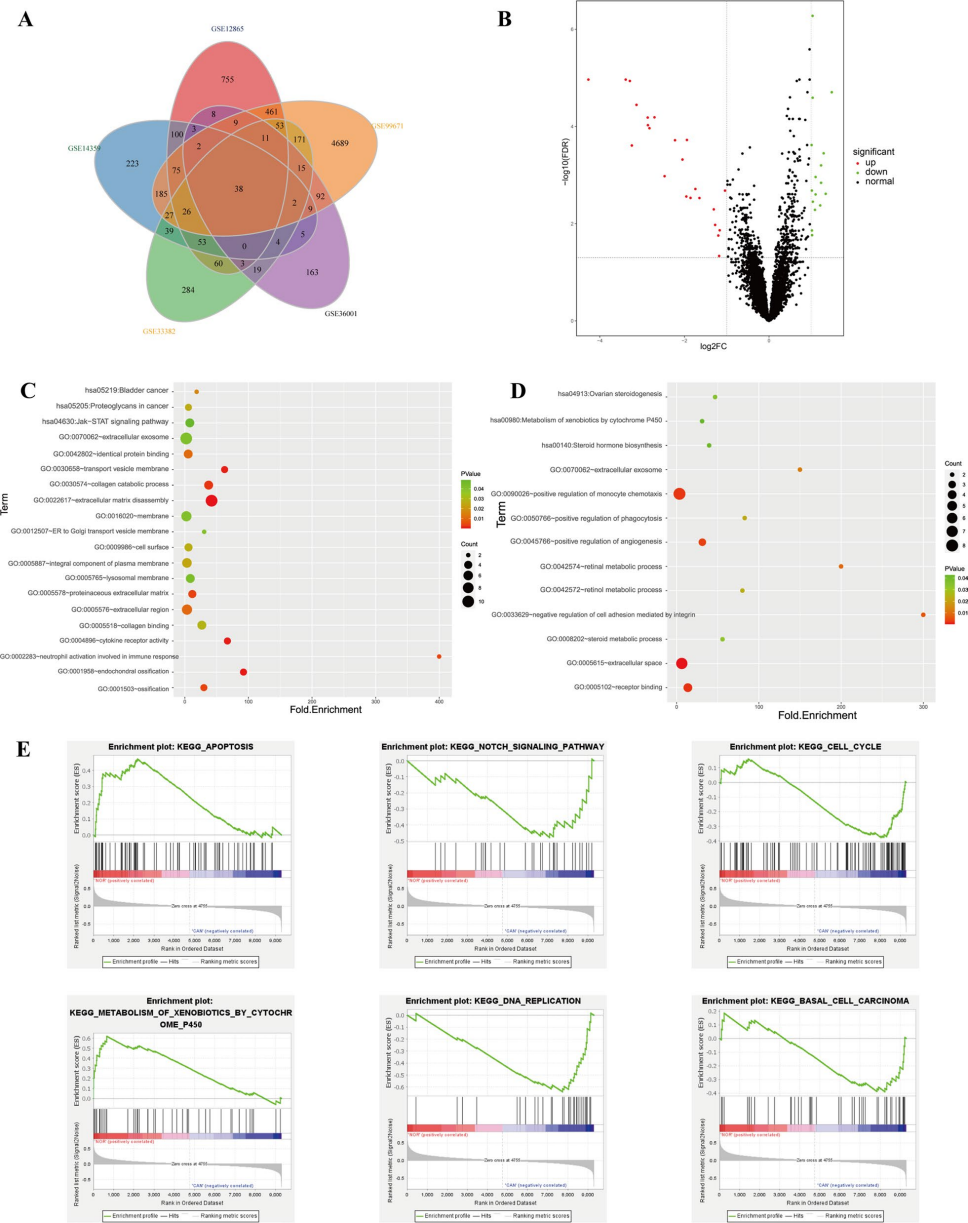
**A** Venn plot of differentially expressed genes among 5 series. **B** Volcano plot to visualize the gene distribution. Red dots represented up-regulated DEGs, green dots represented down-regulated DEGs and black dots represented normal genes. **C** Bubble chart of functional and pathway enrichment analysis of up-regulated genes. **D** Bubble chart of functional and pathway enrichment analysis of down-regulated genes. **E** Gene set enrichment analysis of the whole DEGs

After removing batch effects in these series, we analyzed gene expression of these 5 OS datasets, based on the cutoff criteria |logFC|> 1 and adjusted P-Value < 0.05. In summary, a total of 1632 DEGs were identified in GSE12865, of which 605 up-regulated and 1027 down-regulated; 766 DEGs were in GSE14359, of which 245 up-regulated and 521 down-regulated; 780 DEGs in GSE33382, of which 346 up-regulated and 434 down-regulated; 358 DEGs in GSE36001, of which 203 up-regulated and 155 down-regulated; 5840 DEGs in GSE99671, of which 3365 up-regulated and 2475 down-regulated. Altogether, 38 mutual DEGs were identified by conducting Venn plot analysis to make a more reliable consequence, among them, 23 mutual DEGs were up-regulated and 15 mutual DEGs were down-regulated, together with a volcano plot to visualize the gene distribution (Fig. 2A and B).

### Functional and pathway enrichment analysis on DEGs of OS

To gain further insight and knowledge into the potential functions of genes, the mutual up-regulated and down-regulated genes in these 38 DEGs were uploaded into DAVID database to analyze the significance. The detailed information of GO and KEGG analysis were shown in Fig. 2C–E. GO analysis results revealed that mutual up-regulated DEGs were mainly associated with several biological process (such as extracellular matrix disassembly, endochondral ossification, osteoblast differentiation and collagen catabolic process); cellular components (such as transport vesicle membrane, proteinaceous extracellular matrix, extracellular region) and molecular functions (such as collagen binding and protein binding), while down-regulated DEGs were mainly enriched in cell adhesion, organ regeneration, negative regulation of cell migration and extracellular exosome. KEGG analysis indicated that up-regulated genes were involved in JAK-STAT signaling pathway as well as pathways in proteoglycans of cancer and bladder cancer, while down-regulated DEGs were mostly associated with steroid hormone biosynthesis and metabolism of cytochrome P450. As for the gene set enrichment analysis (GSEA), results indicated that in normal tissues, signaling pathways were primarily associated with apoptosis and metabolism of xenobiotics; while in tumor tissues, pathways were mostly enriched in basal cell carcinoma, cell cycle, DNA replication and notch signaling pathways.

### PPI network construction and hub genes identification

**Fig. 3:**
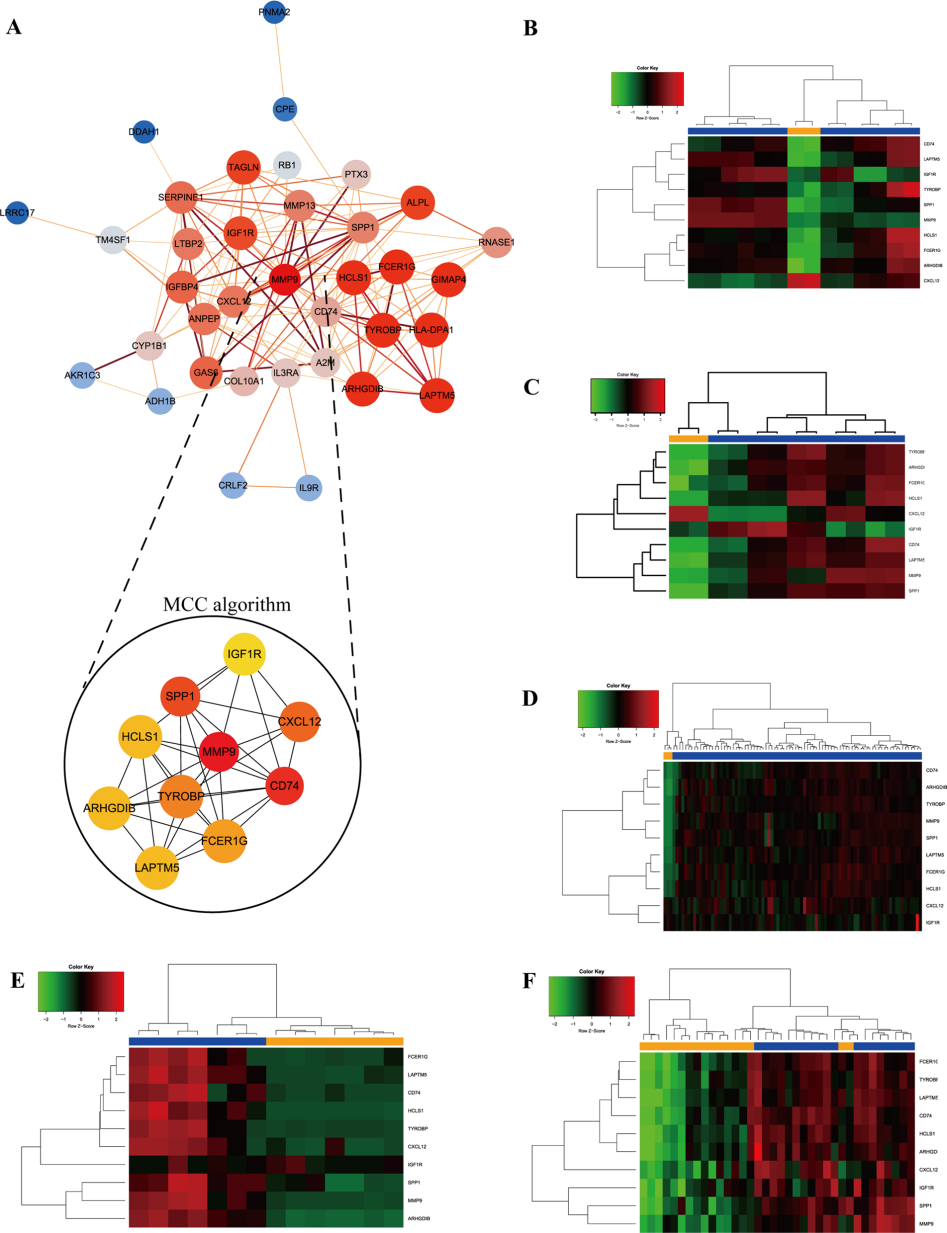
**A** Visualization of the protein–protein interaction (PPI) network as well as the hub genes module calculated by MCC algorithm. **B** Heatmap of hub genes expression in GSE12865. **C** Heatmap of hub genes expression in GSE14359. **D** Heatmap of hub genes expression in GSE33382. **E** Heatmap of hub genes expression in GSE36001. **F** Heatmap of hub genes expression in GSE99671

The protein–protein interaction network of previous 38 mutual DEGs among those 5 series were established by using the STRING database base on P-value cutoff < 0.05, and then the PPI network was imported into Cytoscape for further analysis. Altogether, 36 nodes and 153 edges were generated with PPI network (Fig. 3A). Hub genes were screened by using “cytohubba” plug-in which contained 11 different topological algorithm methods, of which Maximal Clique Centrality (MCC) algorithm was reported to be the most effective method of finding key targets and sub-module from a complexity network. According to the MCC scores of this network, the top ten highest-scored genes, including MMP9, CD74, SPP1, CXCL12, TYROBP, FCER1G, LAPTM5, HCLS1, ARHGDIB, and IGF1R were ultimately identified as hub genes, as listed in Additional file 4: Table S1. Among them, MMP9 ranked highest, suggesting that MMP9 was the most related gene in the development of Osteosarcoma, then this study further focused on MMP9 to find potential inhibitors in structural biology part. Hierarchical clustering analysis in each gene expression profiles demonstrated that these 10 hub genes could significantly distinguish normal samples from tumor samples (Fig. 3B–F).

### The robustness assessment of SVM in gene signatures

To validate the robustness of these 10 genes signature, GSE12865, GSE14359, GSE36001 and GSE99671 were utilized as testing sets to verify the SVM generated by the selected optimized hub genes, five-fold cross validation was conducted during machine learning to obtain the most fitting equation as well as the most accurate results of the testing sets. After training, ROC curve for each testing set was plotted. As shown in Additional file 5: Figure S4, the area under curve (auc) in GSE12865 was 91.67%, auc in GSE14359 was 97.05%, auc in GSE36001 was 77.08% and auc in GSE99671 was 83.33%.

### Validation of gene signature robustness

To validate the robustness of the 10 genes signature, we calculated the RiskScore of the expression level for each sample. The RiskScore value distribution was shown in Additional file 6: Figure S5A, which was statistically significant (P < 0.01). From Additional file 6: Figure S5B, the overall survival time decreased with the risk value increasing. ROC analysis of the prognostic RiskScore classification was then performed based on multivariate Cox analysis, as shown in Additional file 1: Figure S5C, the model had a high AUC value in 3-year prediction (0.776), 5-year prediction (0.759) and 10-year prediction (0.736), respectively. They could make a good prognostic prediction in survival time. Survival analysis of high- and low-risk groups suggested that according to classification of clinical set, 43 patients were scored as low-risk and 43 patients were rated as high-risk. There was significant difference between different risk groups in clinical outcomes, low-risk group had a better survival prognosis compared to high-risk group (log-rank P < 0.0001) (Additional file 6: Figure S5D). Based on the results above, these 10 genes signature could be regarded as risk factors.

### Gene signature model evaluation

**Fig. 4:**
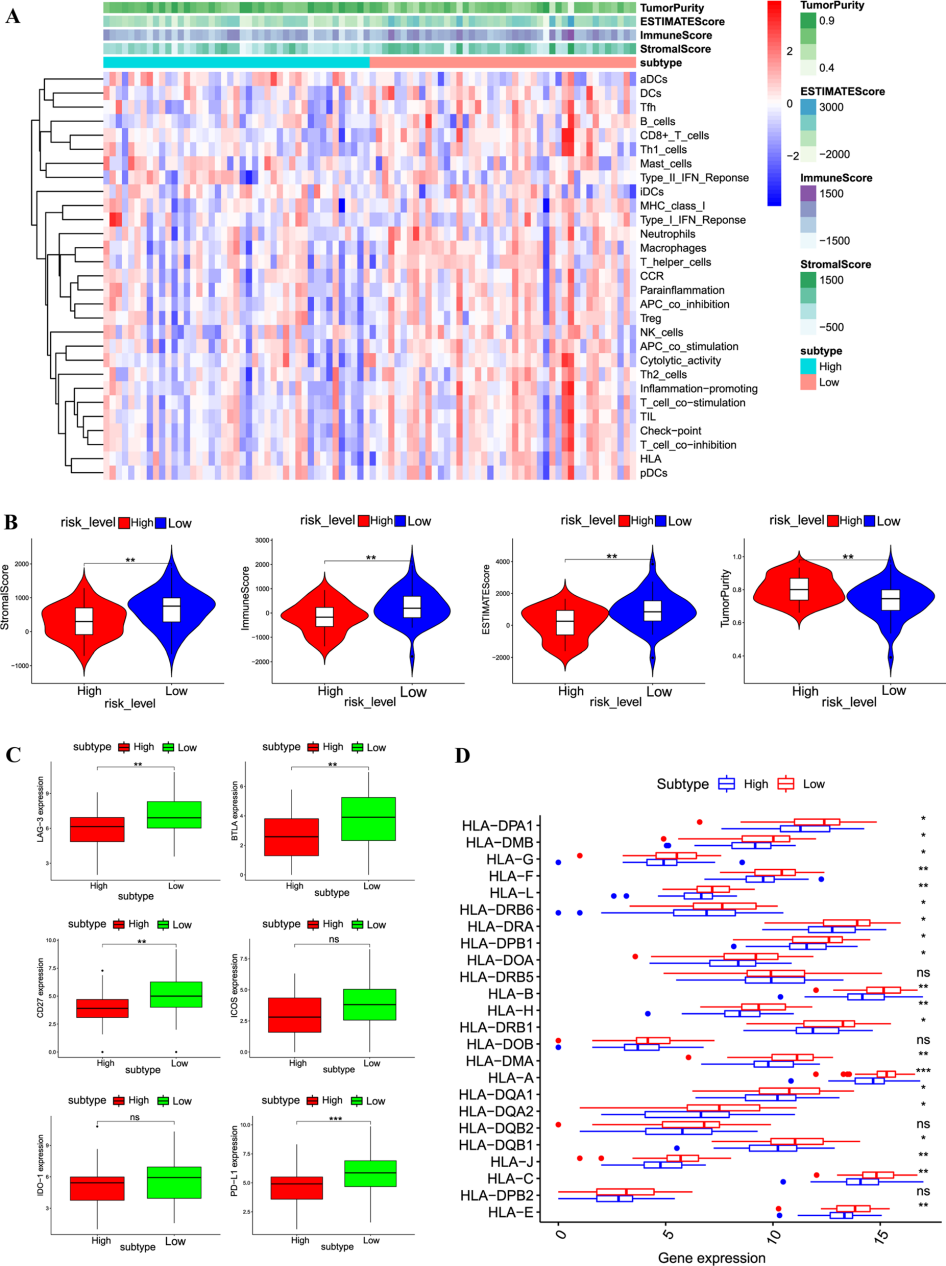
Immunogenomic analysis between high- and low-risk group. **A** The enrichment levels of the 29-immune gene sets by ssGSEA score in each osteosarcoma sample. ESTIMATE method was performed to evaluate tumor purity, stromal score as well as immune score. **B** Comparison of 10-gene signature between high-risk and low-risk group in stromal score, immune score, estimate score and tumor purity. **C** Comparison of immune check point genes expression levels between high-risk and low-risk group. **D** Comparison of expression levels of HLA genes between high-risk and low-risk group. *P < 0.05, **P < 0.01, ***P < 0.0001, ns: no significant difference

After verification of these 10 genes signature’s predictive ability, these 10 genes were regarded as risk factors, so the relationship between RiskScore of the gene signature model and the 29 immune gene sets attracted our interest. Through the ssGSEA method, ssGSEA score [26] was applied to measure the activity or enrichment levels, functions as well as pathways of diverse immune cells in each osteosarcoma samples. According to ssGSEA results, the immune cells, functions and pathways in 29 immune gene sets were mainly enriched in low-risk group (Fig. 4A). Besides, the immune score, stromal score, estimate score and tumor purity were calculated separately for each sample (ESTIMATE method). The result of ESTIMATE depicted that there was significant difference between high and low risk group in terms of immune score, stromal score, estimate score as well as tumor purity. Low-risk group had higher immune score (P < 0.01), stromal score (P < 0.01) and estimate score (P < 0.01) compared to high-risk group, while tumor purity in low-risk group were significantly lower than high-risk group (P < 0.01) (Fig. 4B). In summary, low-risk group contained the most immune cells as well as stromal cells whereas tumor cells were mainly enriched in high-risk group. In addition, we analyzed the expression of HLA genes and immune check point genes between different risk groups, as shown in Fig. 4C, most immune check point genes showed significantly higher expression levels in low-risk group (P < 0.01) except IDO-1 and ICOS. As shown in Fig. 4D, results illustrated that the HLA genes expression in low-risk group was significantly higher than in high-risk group (P < 0.05) except HLA-DRB5, HLA-DOB, HLA-DQB2 and HLA-DPB2.

### Verification of prognostic values of gene signatures by survival analysis

After validating the robustness of hub genes in different GSE datasets by machine learning, this study further verified the prognostic values of hub genes in patients with osteosarcoma in third-party TCGA and GEPIA2 database. Survival curve analysis of overall survival was carried out by Kaplan–Meier plotter using R (“survival” and “survminer” package) in TCGA database. With overall survival as the prognostic outcomes of patients with osteosarcoma, Kaplan–Meier analysis suggested that low-expressed patients showed significantly higher survival time compared to patients with high-expressed hub genes (P < 0.05), except SPP1, LAPTM5 and IGF1R, as shown in Additional file 7: Figure S6. While in GEPIA2 database for analysis with disease free survival (DFS), DFS displayed no significant difference observed in sarcoma patients whether with the high and low expression level of hub genes (Additional file 8: Figure S7). Namely, as for overall survival, patients with low-expression of hub genes showed a significantly favorable prognosis except SPP1, LAPTM5 and IGF1R (P < 0.05), accompanied with a higher percent survival, while saying disease free survival, illustration results could not distinguish the difference of disease-free time between high and low expression level of these hub genes.

### Prediction of overall survival in clinical application

**Fig. 5:**
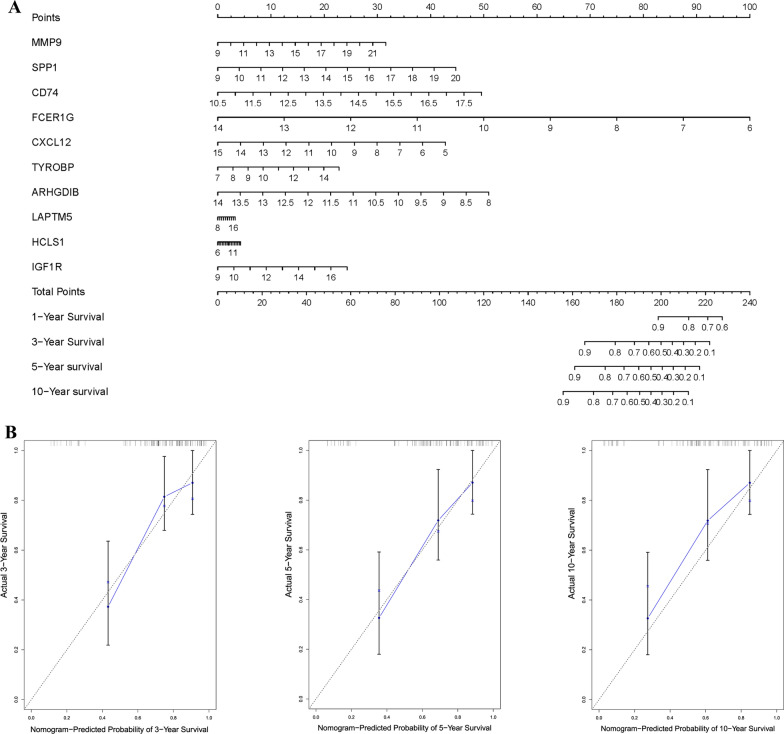
Construction of the nomogram model based on 10-gene signature. **A** Nomogram model for predicting the probability of 3-, 5- and 10-years overall survival in osteosarcoma patients. **B** Calibration plots of the nomogram for predicting the probability of overall survival at 3-, 5- and 10-years

After demonstrating that these 10 genes signature was highly associated with overall survival by multivariate Cox analysis and survival analysis (Additional file 6: Figures S5C, Additional file 7: Figure S6 and Additional file 8: Figure S7). We established the gene signature-based nomogram model (Fig. 5A), the concordance index (C-index: 0.74) showed preferable discrimination ability of the nomogram model. Next, calibration plots of observed vs. predicted probabilities of 3-, 5- and 10-years’ overall survival demonstrated a high concordance, indicating that the model established in this study was highly reliable (Fig. 5B).

### Gene expression patterns of MMP9 in different cancers

In order to provide a solid practical and theoretical basis for the subsequent research of targeted inhibitors, the expression levels of MMP9 in different cancers from GEPIA2 database were observed and analyzed to gain a comprehensive understanding in expression pattern, including SARC (sarcoma), GBM (glioblastoma multiforme), KIRC (kidney renal clear cell carcinoma), LUSC (lung squamous cell carcinoma), COAD (colon adenocarcinoma) and BLCA (bladder urothelial carcinoma). As shown in Additional file 9: Figure S8, results demonstrated that MMP9 expression in GBM, KIRC, LUSC, COAD and BLCA were significantly higher than normal samples, indicating the expression of MMP9 in tumor samples could be differentiated significantly from normal samples (P < 0.01). As for SARC, there was no difference in expression of MMP9 between normal and tumor tissues.

### Virtual screening of natural compounds regarding matrix metallopeptidase 9 (MMP9)

The area of ligand-binding pocket was an essential regulatory site of MMP9, since small molecules binding to this active site could inhibit the function of MMP9 protein and thus prevent MMP9 and its downstream signaling pathways. Consequently, this pocket region was chosen as the docking and reference site. Totally 17,799 purchasable, named and biogenic natural products were obtained from ZINC15 repository, crystal structure of MMP9 was selected as the receptor protein to screen optimal compounds, one effective MMP9 inhibitor JNJ0966 was chosen as the reference ligand to compare pharmacologic properties and binding ability with other compounds. After screening, 10,021 natural compounds were found to be eligible to bind with MMP9 by libdock algorithm. Among those, 2976 compounds had higher libdock scores than the reference ligand JNJ0966 (libdock score: 116.113). The top 20 compounds ranked by libdock scores and the reference ligand JNJ0966 were listed in Additional file 10: Table S2.

### Assessment of pharmacologic and safety properties of compounds

Pharmacologic properties of all the 20 screened ligands as well as reference ligand JNJ0966 were firstly assessed by ADME module of DS4.5, including properties of aqueous solubility, blood–brain barrier penetration, human intestinal absorption level, cytochrome P4502D6 (CYP2D6) inhibition, hepatotoxicity and plasma protein binding level. As shown in Additional file 11: Table S3, the aqueous solubility level showed that 10 compounds had a good solubility in water (defined as scores ≥ 3, and water at 25 ℃), which had a better solubility than JNJ0966 (solubility level: 2); all compounds were predicted with high permeability with blood–brain barrier; 17 compounds had a better intestinal absorption level (score: 3) than JNJ0966 did (score: 2). All compounds but ZINC000004654845 were found to be non-inhibition with CYP2D6, which was an important enzyme in drug metabolism. For hepatotoxicity, 14 compounds were predicted with non-toxicity regarding liver whereas JNJ0966 was predicted to be liver-toxicity drug. Finally, plasma protein binding properties indicated that 7 compounds had stronger binding force than JNJ0966 did.

Safety should be also fully assessed when selecting drug candidates. To assess the safety of the 20 compounds, different kinds of indicators among natural compounds were carried out using a computational method in TOPKAT module of DS4.5, such as ames mutagenicity, FDA carcinogenicity level (male rat, female rat, male mouse, female mouse), as well as weight of evidence carcinogenicity. The detailed information parameters of these compounds were shown in Additional file 12: Table S4. For the reference ligand JNJ0966, it was calculated to have high probability in ames mutagenicity and FDA carcinogenicity whether in rat or mouse, except one parameter that in male mouse it was calculated with non-carcinogenicity. In addition, the weight of evidence carcinogenicity in JNJ0966 was predicted with high probability, together with a high probability of developmental toxicity potential. Considering all the results mentioned above, ZINC000072131515, ZINC000085810532, ZINC000014233122, ZINC000030731360 and ZINC000004228235 were further selected as safe drug candidates with low ames mutagenicity and probability of FDA carcinogenicity, non-weight evidence probability of carcinogenicity. Moreover, these candidate drugs had pretty pharmacologic properties such as high solubility level in water, good intestinal absorption, non-hepatotoxicity and non-CYP2D6 inhibition. As a result, these 5 drug candidates were selected at this time and pooled for subsequent research.

### Highly precise docking analysis and visualization

**Fig. 6:**
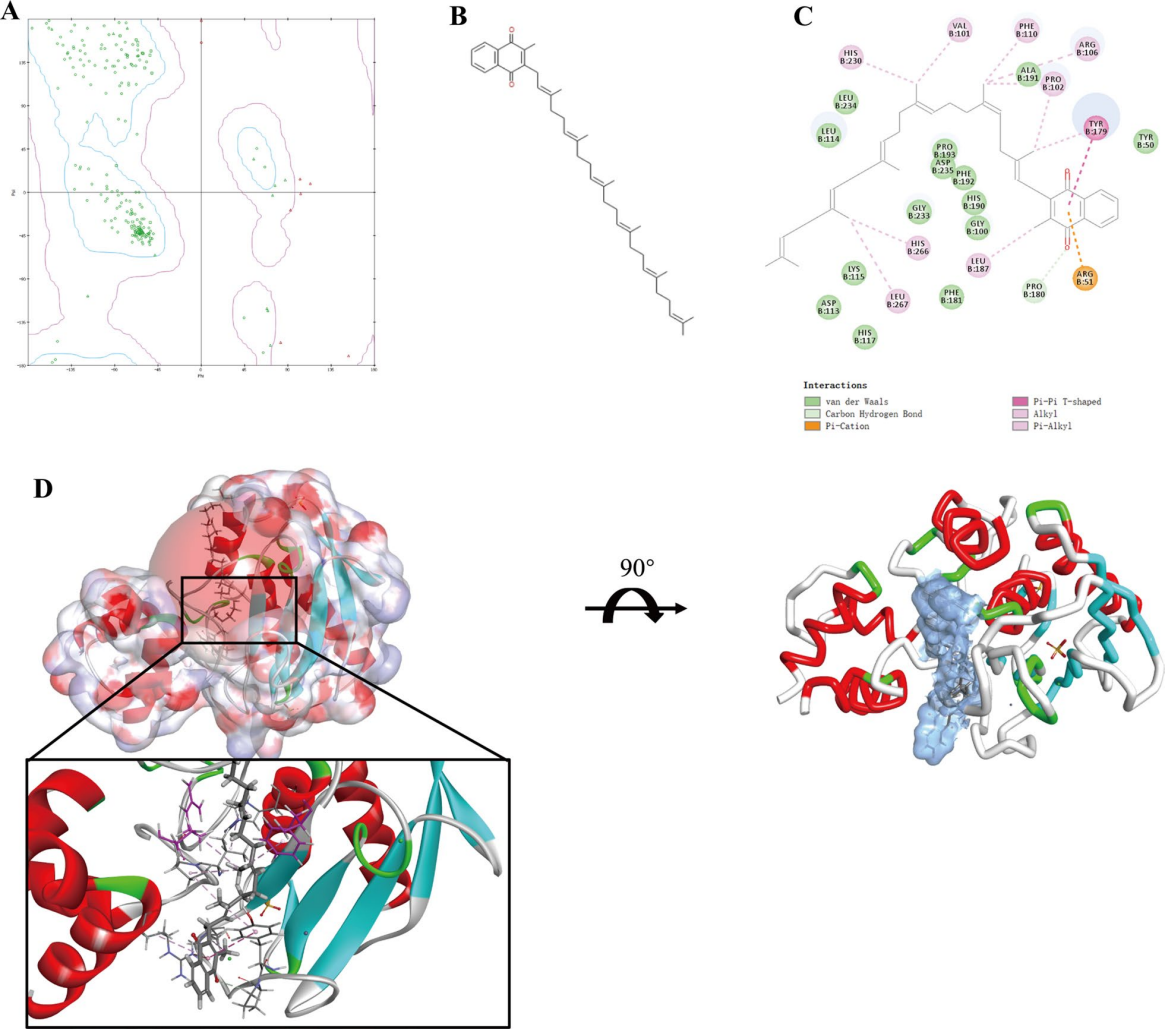
**A** The Ramachandran diagram of MMP9 protein. **B** Chemical structure of novel compound ZINC000072131515 selected from virtual screening. **C** Schematic drawing of inter-molecular interactions of the computed binding modes of ZINC000072131515 with MMP9 based on highly precise docking method. **D** Visualization of chemical interactions between the ligand and MMP9 after highly precise docking (ZINC000072131515-MMP9 complex). The surface of binding area as well as the active binding region were added. Blue represented positive charge, red represented negative charge and active binding region was shown with red sphere. Inhibitors was displayed with sticks, with the structures around ligand-receptor junction shown in thinner sticks

In order to study the ligand binding mechanisms of these selected compounds and JNJ0966 with receptor MMP9, these 5 compounds and JNJ0966 were docked into the ligand-binding pocket of MMP9 by CDOCKER module, which was a high-precision docking program to study the chemical bonds generated between ligands and MMP9. The RMSD between the docked ligand-MMP9 poses and initial crystal structure was 0.4593 Å, proving that the CDOCKER module conducted in this study was highly reliable for reproducing the experiment results. CDOCKER interaction energy between ligands and MMP9 were calculated and displayed in Additional file 13: Table S5. Among them, ZINC000014233122, ZINC000030731360 were failed to generate conformations with MMP9. As for the rest compounds, the CDOCKER interaction energy of ZINC000072131515-MMP9 complex (-55.6816 kcal/mol), ZINC000004228235-MMP9 complex (− 56.348 kcal/mol) and ZINC000085810532-MMP9 complex (− 62.1737 kcal/mol) were much lower than the reference JNJ0966-MMP9 complex (− 37.6049 kcal/mol). Chemical bonds analysis such as hydrogen bonds and others generated between ligands and MMP9 were performed through computational method to visualize the inter-molecule interactions between them (Fig. 6, Additional file 14: Figure S9 and Additional file 15: Figure S10). Results illustrated that ZINC000072131515 formed 1 pair of carbon hydrogen bond, 1 pair of Pi-Cation bond, 6 pairs of alkyl bonds, 5 pairs of Pi-Alkyl bonds, 1 pair of pi–pi interaction and several van der Waals force with MMP9; ZINC000004228235 totally formed 11 pairs of chemical bonds, including 6 pairs of hydrogen bonds, 2 pairs of carbon hydrogen bonds, 2 pairs of Pi-alkyl bonds and 1 pair of Pi-cation bond. ZINC000085810532 only formed 2 pairs of carbon hydrogen bonds, 1 pair of hydrogen bond and 1 pair of Pi-alkyl bond. As for the reference ligand JNJ0966-MMP9 conformation, it formed 12 bonds including 1 Pi-cation bond, 1 Pi-sigma bond, 1 Amide-Pi stacked bond, 5 pairs of Pi-alkyl bonds, 2 pairs of alkyl bonds, together with 2 pairs of unfavorable acceptor-acceptor bonds. The detailed chemical bonds information of ligand-MMP9 complexes was shown in Additional file 16: Table S6.

### ZINC000072131515 inhibited migration and suppressed proliferation of OS cells

**Fig. 7:**
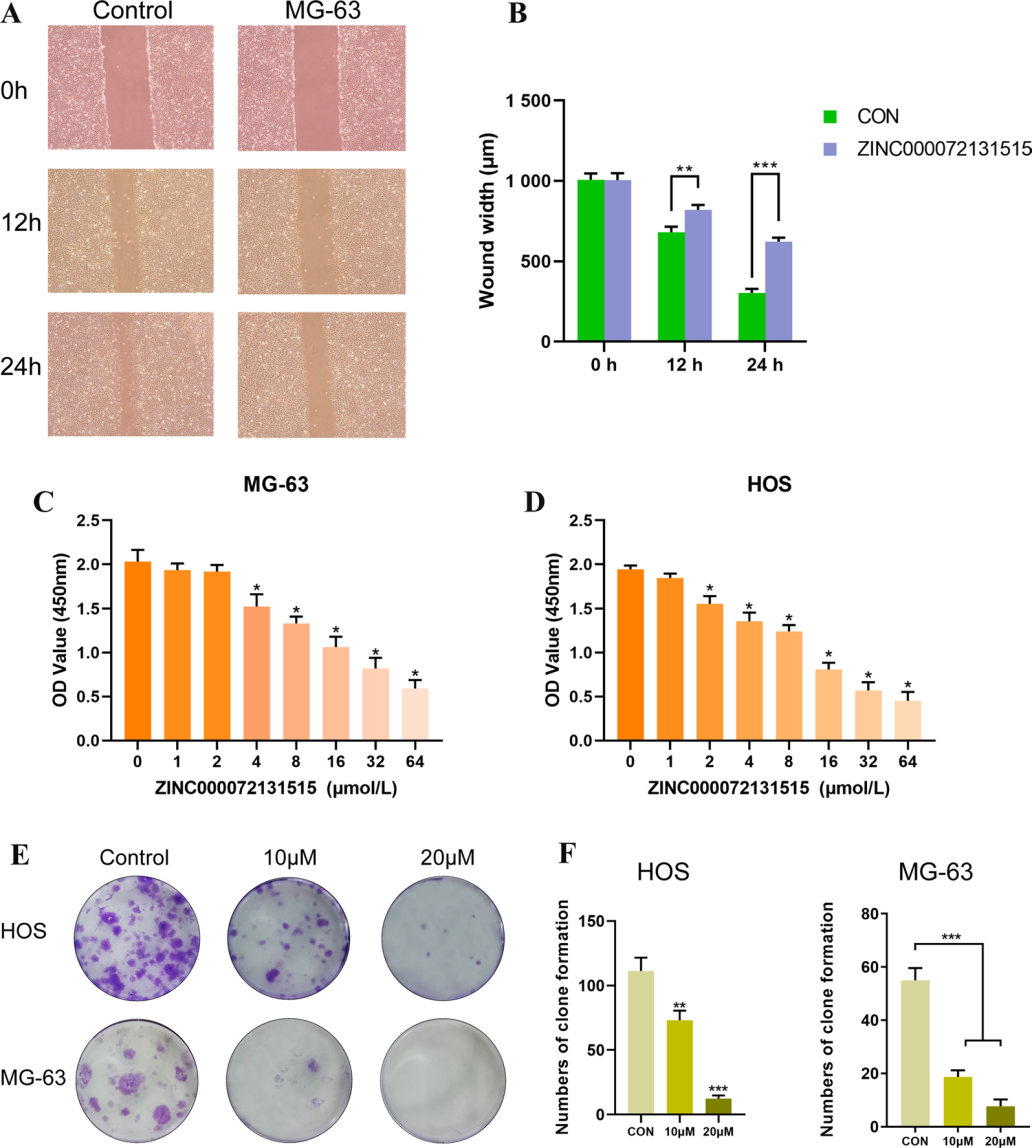
**A** Scratch assay in control and drug group. **B** Wound width in control and drug group. **C**, **D** Cellular viability of osteosarcoma cell lines treat with different doses of drug ZINC000072131515. **E** Clonogenicities in Petri dishes with different doses of drug. **F** Numbers of clone formation in HOS and MG-63 cell lines

In the scratch assay, the width of scratched areas were measured after 0, 12 and 24 h of scratch, to analyze the ability of drugs to influence migration of OS cells. As shown in Fig. 7A, B, the scratch width represented the migration capacity of OS cells, results indicated that the widths in drug group were significantly broader than in control group at 12, 24 h (P < 0.05).

To evaluate the sensitivity of OS cells to drug ZINC000072131515, the survival cells after different doses of drug treatment were calculated by CCK-8, and growing ability was assessed by colony formation assay. OS cell lines were treated with different doses of drug for 48 h (0, 1, 2, 4, 8, 16, 32, 64 μmol/L). As Fig. 7C, D showed, the cellular viability of HOS and MG-63 cells were declined slowly with the increase of drug concentration, when the drug concentration came to 4 μmol/L, the cellular viability were decreased significantly. Results of colony formation assay also suggested that after 2 weeks cultivation, both HOS and MG-63 cell lines showed fewer and smaller clonogenicities in petri dishes with drug compared to the control group (Fig. 7E), and the numbers of clone formation in drug groups was lower than that in control group significantly (Fig. 7F).

## Discussion

Osteosarcoma (OS) is the most common primary malignant tumor in orthopaedics, which mainly occurs in teenagers as well as young adults [9, 31]. Although the treatment of OS including surgical technique and chemotherapy has improved, the overall prognosis of OS has still remained poor [4]. The reason caused this situation may be blamed for lack of precise molecular targets. To the best knowledge, current researches mainly focused on the mechanisms causing metastasis of OS, while the study on molecular targets causing the initiation and progression of OS as well as chemotherapy on targeted genes had not been fully investigated. Therefore, more knowledge about the mechanism in carcinogenicity of OS is imperative for early diagnosis as well as the treatment.

Recent years, microarray technology combined with bioinformatics method have been widely conducted to identify genetic changes at the genome level [18, 32], making it possible for us to identify real hub genes as well as function and pathway alterations responsible for the initiation and development of OS. In addition, structural biology method such as virtual screening, molecular docking, had been commonly applied in drug screening and pharmaceutical chemistry. Structural biology study had made remarkable contribution to medication screening and improvement in treatment of different disease [33–36]. As a result, bioinformatics combined with structural biology analysis were carried out in this study, to make a comprehensive understanding from the progression to the treatment of OS, and assessed properties of the existed inhibitor JNJ0966 regarding MMP9 in the meantime.

In this study, totally 31 normal samples and 132 tumor samples of OS were extracted from 5 different microarray chips in GEO database, aiming to avoid clinical and race bias among different studies and make a convincible result of our research. “Batch effects” existed in those gene expression profiles were firstly eliminated. Here, the terminology “batch” referred to microarrays processed at one location over a short period of time using the same platform, while the cumulative error introduced by these time and place-dependent experimental variations was referred to as “batch effects”, which could affect the results of different experiments and finally confound or mask the real biological meaning among different datasets [37, 38]. Meanwhile, it could be much complicated to the unified preprocessing and analysis of different data. In order to prove the existence of batch effects in this study as well as outcome after removing batch effects, principle component analysis (PCA) was conducted and results were plotted to visualize. Illustrations showed that before eliminating batch effects, different series were clustered at different place, which meant these 5 series had different expression pattern due to batch effects. After eliminating, the points which represented different series were clustered with each other, with non-difference in point distribution on image. PCA provided a solid evidence for both existence and elimination of batch effects. Subsequently, PCA was applied again to reduce dimension among these 5 series based on processed matrix (normal versus tumor), results demonstrated these two phenotypes could be significantly differentiated from each other, elucidating that there was a real biological difference, and thus offered a solid basis for our following research.

A total of 1632 DEGs, 766 DEGs, 780 DEGs, 358 DEGs and 5840 DEGs were identified, respectively from those 5 series, with the cutoff criteria of |logFC|> 1 and adjusted P-value < 0.05. Venn plot analysis was performed further to obtain precise results, and 38 mutual DEGs were generated. After GO analysis of these mutual DEGs, we detected that those up-regulated genes were mainly associated with collagen catabolic process, extracellular matrix disassembly, osteoblast differentiation, extracellular region and protein binding, which explained why the fast multiplication and invasion of tumor cells, these findings agreed with previous studies that extracellular matrix (ECM) played a pivotal role in tumor metastasis and invasion [39, 40]. Down-regulated genes were primarily involved in cell adhesion, organ regeneration and negative regulation of cell migration, which could slow the tumor invasion as well as promote the growth of normal tissues. Furthermore, the analysis of KEGG displayed that these mutual up-regulated DEGs were mainly enriched in JAK-STAT signaling pathway as well as proteoglycans in cancer. Extracellular matrix (ECM) and substrate constitute the first barrier of tumor metastasis, the major component of the ECM are fibrous proteins (such as collagens, elastins) and proteoglycans, which was activated aberrantly in the development of cancer [41]. In addition, GSEA results showed that cell cycle, DNA replication and notch signaling pathways were aberrantly activated in osteosarcoma patients. Advanced studies have reported that dysregulation of cell cycle process played an pivotal role in the carcinogenesis of tumor [42–44]; DNA replication was the most vulnerable cellular process which could lead to cancer [45]; and notch signaling pathway was reported to play a critical role in skeletal development as well as homeostasis, and serious skeletal disorders could be blamed for alterations in notch signaling [46].

With the aim of further screening hub genes from DEGs identified in our previous work, the mutual 38 DEGs were analyzed with the construction of PPI network based on STRING database. MCC algorithm from “cytohubba” plug-in in Cytoscape was applied to calculate MCC scores from different mutual genes, and the top 10 related-OS genes were screened out (namely MMP9, CD74, SPP1, CXCL12, TYROBP, FCER1G, LAPTM5, HCLS1, ARHGDIB, and IGF1R). Among them, MMP9 ranked highest in MCC algorithm, which was reported to be the most effective method to identify key targets and sub-network in a complexity network, suggesting that MMP9 was the most related gene in the development of Osteosarcoma, so this study further focused on MMP9 to find potential inhibitors in structural biology part. Hierarchical clustering in each expression profiles validated that these hub genes could significantly distinguish normal samples from tumor samples, in the meantime, their expression patterns were found to be up-regulated in tumor tissues of OS compared with normal tissues.

In order to prove the robustness of these 10 hub genes signature, one of the machine learning methods Support Vector Machine (SVM), was conducted to train the predictions with GSE33382 setting as training set, together with five-fold cross validation. This 10 genes signature was validated by robustness as well as estimation worth, by comparing SVM model to other independent datasets. ROC curve of each testing set was plotted, which was used to reflect the relationship between sensitivity and specificity and judge whether a certain factor had diagnostic value for a disease. The area under curve (auc) in GSE12865, GSE14359, GSE36001, GSE99671 was 91.67%, 97.05%, 77.08% and 83.33%, respectively, elucidating that these hub genes could make accurate predictions on the phenotype of unknown tissue, which could be applied in clinical diagnosis. Subsequently, RiskScore was calculated for each sample through multivariate Cox analysis to further confirm the reliability of these genes signature, ROC curve illustrated that high- and low-risk group categorized by these genes signature could make a preferable prognostic classification for 3-, 5- and 10-years overall survival, high-risk group had less survival time compared to low-risk group, Kaplan–Meier analysis also demonstrated that low-risk group patients had a better prognosis than high-risk group. The results mentioned above implied that these 10 genes signature could be regarded as risk factors, consequently, the significance of these genes were confirmed again in the development of osteosarcoma.

After validating the robustness of these genes signature, we wonder whether RiskScore of genes signature model had relationship with immune system microenvironment. It’s worth mentioning that through ssGSEA method, immune cells and functions were mainly enriched in low-risk group, and tumor purity were mostly in high-risk group. This study further analyzed the HLA-genes activity in different risk groups, results showed that HLA genes (except HLA-DRB5, HLA-DOB, HLA-DQB2 and HLA-DPB2) mainly participated immunoregulation in low-risk group. These findings elucidated the high correlations between risk groups and immune microenvironment, immune check point inhibitors may be more effective for patients with high immune check point expression. More detailed mechanisms between these genes signature and immunoregulation need to be analyzed in further study.

Then, survival analysis about overall survival and disease-free survival (DFS) were carried out to further verify the clinical significance and prognostic values of these hub genes in third-party database. Kaplan–Meier analysis illustrated that OS patients with low expression of these genes showed a better prognosis in overall survival (P < 0.05) except SPP1, LAPTM5 and IGF1R, which may be blamed for the relatively low accuracy of SVM classifier in GSE36001 (77.08%). As for disease-free survival, DFS displayed no difference observed in sarcoma patients between high and low expression value of these genes. These findings implied that the overall survival prognosis of patients with OS could be predicted in clinical by detecting the expression level of those hub genes. While in the aspect of DFS, the expression value of these genes could not prolong or shorten the disease-free time of patients, elucidating that this disease was easy to relapse and dangerous, hub genes identified in this study were much essential, diagnosis and treatment should be performed at early stage in order to make a good prognosis. Based on our previous findings, this study established the 10-gene signature-based nomogram model, thus the approximate survival rate in 1-, 3-, 5- and 10-years of osteosarcoma patients could be predicted by detecting these 10 genes’ expression in clinical, calibration plots and C-index (0.74) could confirm the reliability of this nomogram model. Therefore, this nomogram model could allow surgeons to make a comprehensive assessment of patients’ prognosis and thus make appropriate treatment in clinical application.

Based on the results of MCC algorithm scores in Cytoscape, MMP9 ranked highest among those hub genes, suggesting that MMP9 may be the most related gene that caused the progression of osteosarcoma, and it was meaningful to focused on MMP9 for subsequent research. To further assess the significance as well as importance of MMP9 to make a comprehensive understanding, we evaluated the expression pattern of MMP9 in different cancers from GEPIA2 database, such as SARC, GBM, KIRC, LUSC, COAD and BLCA. Results showed that the median expression value of these tumors were much higher than normal tissues (P < 0.01), which suggested that MMP9 was an essential biomarker that could also be diagnosed in many other cancer types, so it provided a solid practical and theoretical basis on the subsequent research of targeted chemotherapy regarding MMP9 in this study. The median expression level of MMP9 in SARC showed no difference between normal and tumor tissues, which may be blamed for small sample size of normal tissues in GEPIA2, however, this study filled up the gap of MMP9 research in SARC, MMP9 was highly expressed in osteosarcoma patients. It is noteworthy that not only MMP9 could be served as therapeutic target, but other related hub genes identified in this study have these potentials for inhibitors design to prevent the development of Osteosarcoma.

MMP9, located on chromosome 20q11.1 ~ 13.1, has 13 exons and 9 introns, which belongs to matrix metalloproteinases family (MMPs). MMPs are the main enzymes for degradation of ECM, these enzymes have low activity in normal conditions, but their activities are increased aberrantly in pathological conditions such as tumors [40]. The MMP family could degrade ECM proteins, ECM degradation is a characteristic of tumors in tumor progression [47, 48]. Consequently, on the basis that MMP9 was the most related gene in OS, as well as its significance in other cancer types, screening of inhibitors targeting MMP9 was urgent and conducted in the following research.

At present, the chemotherapeutics to treat osteosarcoma had been less marked, survival rates continued to be unsatisfactory [4]. The purpose of the treatment is to alleviate disease progression, improve life quality, prolong the survival time in a way which does the least harm to patients. Several decades of intensive investigation have not yielded therapeutically viable MMP inhibitors, which has been attributed to the generally poor specificity of active site of MMP inhibitors, causing dose-limitation toxicities as well as adverse side effects [12]. Only several studies have reported the reliability in discovery of MMP9 inhibitors, and made a significant advance in the field of MMP9 inhibition [11, 12]. Thus, more potential lead compounds regarding MMP9 were imperative for research, to provide more novel skeleton of candidates in pharmaceutical market. Meanwhile, this study also assessed the properties of the discovered inhibitor JNJ0966.

In subsequent study of structural biology, 17,799 purchasable, named and biogenic natural products were obtained from ZINC15 repository to screen targeted inhibitors of MMP9. This study combined with ADME, TOPKAT, CDOCKER, and other computational methods in DS4.5, to make a fully screening and assessment of these natural compounds as well as the existed inhibitor JNJ0966. Virtual screening using Libdock module was firstly conducted in order to screen appropriate compounds docked with MMP9 from huge amount of ligands repository, which allowed us to narrow the range of candidate drugs in a short time. Libdock scores represented the energy optimization and stability of the conformation. Compounds with higher libdock scores illustrated a better energy optimization as well as a more stable conformation. After calculated by virtual screening, totally 10,021 products could dock at ligand-binding pocket of MMP9, among those, 2976 products’ libdock scores were found higher than the reference ligand JNJ0966 (score: 116.113), indicating that these 2976 products had a more stable conformation together with a better energy optimization with receptor MMP9 compared to JNJ0966. On the basis of libdock scores, the top 20 highest compounds were selected and pooled into further study.

Pharmacological properties including ADME (absorption, distribution, metabolism and excretion) and toxicity predictions for those obtained candidate drugs were conducted to select drugs which had good pharmaceutic properties. Outcomes indicated that after overall assessment of their properties as well as safeties, 5 compounds: ZINC000072131515, ZINC000085810532, ZINC000014233122, ZINC000030731360 and ZINC000004228235 were selected as efficient drugs with good water solubility, good intestinal absorption level and strong plasma protein binding. Moreover, they were predicted with non-hepatotoxicity and non-inhibition of CYP2D6, which could reduce the damage to liver whereas JNJ0966 was predicted to be toxic drug to liver. Cytochrome p450 (CYP450) was a principal enzyme involved in drug metabolism, drugs behaved as inhibitors of CYP450 could weaken the activity of drug enzymes and thus slow down the drug metabolism. In most cases, it leaded into enhancement of pharmacological activity of targeted drugs or even toxic side effects, which was shown to be frequent and dangerous one [49, 50]. CYP2D6 is one of the enzymes in CYP450, thus the result of non-inhibition of CYP2D6 of these compounds proved that they were safe and metabolizable drug candidates, the same as JNJ0966. Additionally, they were assessed with low ames mutagenicity, low probability of FDA carcinogenicity and non-weight evidence probability of carcinogenicity compared to JNJ0966, which strongly suggested their perspective application in drug development. Consequently, these 5 compounds were further selected as candidate drugs and conducted in subsequent research.

Docking mechanisms and chemical bonds interactions of these compounds with MMP9 were then analyzed and visualized using precisely docking method. Of which, ZINC000014233122, ZINC000030731360 were failed to dock at the ligand-binding pocket of MMP9, which indicated that these two ligands could not be able to form stable chemical bonds with MMP9, more stronger bonds were needed in CDOCKER module. CDOCKER interaction energy of the rest of three compounds with MMP9 had a significant lower energy compared to the reference ligand JNJ0966 (− 37.6.49 kcal/mol), which demonstrated that they had a better binding affinity with MMP9. Analysis of chemical bonds interactions showed that ZINC000085810532 did not form as many bonds as ZINC000072131515, ZINC000004228235 and JNJ0966 did. Taking all the evaluation indexes into consideration, ZINC000072131515 and ZINC000004228235 were ultimately chosen as efficient lead compounds regarding MMP9. The overall detailed chemical parameters between these two compounds and MMP9 were conducted with an intuitive visualization (Fig. 6 and Additional file 14: Figure S9).

In order to prove the effects of our newly found compounds against osteosarcoma, this study further performed a series of experiments in vitro regarding ZINC000072131515. Scratch assay revealed that the wounds in control group decreased more sharply than drug group with time went, which implied that the migration ability of osteosarcoma cells was restrained by ZINC000072131515. Moreover, CCK-8 and colony formation assay pointed that the cellular viability in HOS and MG-63 cell lines displayed dose-dependent decreased when treated with different concentration of drug ZINC000072131515. In comparison to control group, clonogenicities of drug group were less and smaller significantly, which were consistent with CCK-8 assay, these results elucidated that ZINC000072131515 inhibit the proliferation of osteosarcoma cells.

Last but not least, this study tried to find novel natural inhibitors through an effective and rigorous way, it’s worth noting that no single drug, could be marketed directly unless through thousands of refinements and modifications of the candidate drugs, together with countless animal and cell experiments. The two compounds identified in this study provided a basis and skeleton for inhibitors of MMP9, further research could focus on refinement and improvement of them by adding or deleting different groups of atoms or pharmacophores in order to further reduce toxicity, promote pharmacologic properties. These two candidate compounds offered a valuable resource for the development of MMP9-related inhibitors.

At the present time, identifying hub genes from different cancer types under genomic level and development of oncology drugs are the hot research attracting worldwide attention [13, 14, 51, 52]. This study elucidated the procedure from identification of hub genes in osteosarcoma, to the study of inhibitors targeting MMP9 in detail, each step was clearly explained. Meanwhile, this study made an overall evaluation of expression pattern of MMP9 in different cancer, and filled up the gap in research of MMP9 in osteosarcoma. Different pharmacophores could be added to improve the properties of these compounds in the following research, after modification, these compounds could be more perfect as potential inhibitors. Besides, the application of MMP9 inhibitor regarding osteosarcoma had not been reported so far, so further research in this field could make a remarkable significance in the chemotherapy of osteosarcoma.

## Conclusions

In conclusion, 10 main hub DEGs were identified from 5 GSE series, namely MMP9, CD74, SPP1, CXCL12, TYROBP, FCER1G, LAPTM5, HCLS1, ARHGDIB, and IGF1R. The robustness, prognostic values as well as immuno-correlation of these genes were validated with machine learning, multivariate Cox analysis, ssGSEA and survival analysis. This study found MMP9 was the most related hub gene in the progression of osteosarcoma. Meanwhile, this study filled the gap in research of MMP9 in osteosarcoma. Structural biology study including a series of computation-aided methods demonstrated that ZINC000072131515 and ZINC000004228235 had the effective potential inhibition targeting MMP9, which were selected as candidate and promising drugs. Ultimately, it may have great significance in MMP9 inhibitors’ development.

## Supplementary Information

**Additional file 1: Figure S1.** Crystal structure of Matrix metalloproteinase-9 (MMP-9). (A), Initial crystal structure. (B), Bing surface added. Blue represented positive charge and red represented negative charge.

**Additional file 2: Figure S2.** Boxplot of normalized unscaled standard errors (NUSE) of (A) GSE12865 and (B) GSE14359, which were used for quality control. Boxplot or violin plot after matrix background correction and normalization of (C) GSE12865; (D) GSE14359; (E) GSE33382; (F) GSE36001 and (G) GSE99671, which were displayed to visualize and verify the expression distribution.

**Additional file 3: Figure S3.** (A), PCA scatter plot among 5 series before removing “batch effects”. PCA: principal component analysis. (B), PCA scatter plot among 5 series after eliminating “batch effects”. (C), Generated QQ-plot and density plot after eliminating “batch effects”. (D), PCA analysis between different phenotypes based on the processed matrix, normal tissues could be distinguished from tumor tissues on PCA1 axis.

**Additional file 4: Table S1.** Detailed score information of hub genes through MCC algorithm.

**Additional file 5: Figure S4.** Area under curve of ROC diagram of support vector machine (SVM).

**Additional file 6: Figure S5.** Construction of risk group base on RiskScore by multivariate Cox analysis. (A), Scatter plot of risk scores distribution in high-risk and low-risk group. (B), Overall survival time with different risk scores. (C), ROC curve of 10-gene signature for prognostic classification. (D), K-M survival prognosis of 10-gene signature model.

**Additional file 7: Figure S6.** Kaplan–Meier overall survival analysis of 10 hub genes in osteosarcoma patients from TCGA database. (A), Survival analysis of MMP9 in osteosarcoma. (B), Survival analysis of CD74 in osteosarcoma. (C), Survival analysis of SPP1 in osteosarcoma. (D), Survival analysis of CXCL12 in osteosarcoma. (E), Survival analysis of TYROBP in osteosarcoma. (F), Survival analysis of FCER1G in osteosarcoma. (G), Survival analysis of LAPTM5 in osteosarcoma. (H), Survival analysis of HCLS1 in osteosarcoma. (I), Survival analysis of ARHGDIB in osteosarcoma. (J), Survival analysis of IGF1R in osteosarcoma.

**Additional file 8: Figure S7.** Disease-free survival (DFS) analysis of 10 hub genes in osteosarcoma patients from the GEPIA2 database. (A), Survival analysis of MMP9 in osteosarcoma. (B), Survival analysis of CD74 in osteosarcoma. (C), Survival analysis of SPP1 in osteosarcoma. (D), Survival analysis of CXCL12 in osteosarcoma. (E), Survival analysis of TYROBP in osteosarcoma. (F), Survival analysis of FCER1G in osteosarcoma. (G), Survival analysis of LAPTM5 in osteosarcoma. (H), Survival analysis of HCLS1 in osteosarcoma. (I), Survival analysis of ARHGDIB in osteosarcoma. (J), Survival analysis of IGF1R in osteosarcoma.

**Additional file 9: Figure S8.** MMP9 expression patterns in different cancer types including SARC, GBM, KIRC, LUSC, COAD, BLCA. SARC: sarcoma; GBM: glioblastoma multiforme; KIRC: kidney renal clear cell carcinoma; LUSC: lung squamous cell carcinoma; COAD: colon adenocarcinoma; BLCA: bladder urothelial carcinoma. *P < 0.01.

**Additional file 10: Table S2.** The top 20 ranked compounds with higher libdock scores as well as the reference compound JNJ0966.

**Additional file 11: Table S3.** ADME (absorption, distribution, metabolism and excretion) properties of the top 20 compounds.

**Additional file 12: Table S4.** Toxicity predictions of the top 20 compounds.

**Additional file 13: Table S5.** CDOCKER interaction energy of compounds with Matrix metalloproteinase-9 (MMP-9).

**Additional file 14: Figure S9.** (A), Chemical structure of novel compound ZINC000004228235 selected from virtual screening. (B), Schematic drawing of inter-molecular interactions of the computed binding modes of ZINC000004228235with MMP9 based on highly precise docking method. (C), Visualization of chemical interactions between the ligand and MMP9 after highly precise docking method (ZINC000004228235-MMP9 complex). The surface of binding area as well as the active binding region were added. Blue represented positive charge, red represented negative charge and active binding region was shown with red sphere. Inhibitors was displayed with sticks, with the structures around ligand-receptor junction shown in thinner sticks.

**Additional file 15: Figure S10.** (A), Schematic drawing of inter-molecular interactions of the computed binding modes of ZINC000085810532 with MMP9 based on highly precise docking method. (B), Chemical structure of the reference ligand JNJ0966. (C), Schematic drawing of inter-molecular interactions of the computed binding modes of the reference ligand JNJ0966 with MMP9 based on highly precise docking method. (D), Visualization of chemical interactions between the reference ligand and MMP9 after highly precise docking method (JNJ0966-MMP9 complex). The surface of binding area as well as the active binding region were added. Blue represented positive charge, red represented negative charge and active binding region was shown with red sphere. Inhibitors was displayed with sticks, with the structures around ligand-receptor junction shown in thinner sticks.

**Additional file 16: Table S6.** Chemical bond interaction parameters of each compound with MMP9 residues.

## Data Availability

The data used and analyzed in this study are available upon reasonable request.

## Abbreviations

DAVID: Database for annotation, visualization, and integrated discovery
DS: Discovery Studio (version: 4.5)
FDA: Food and drug administration
MMP-9: Matrix metalloproteinase-9
OS: Osteosarcoma
RMSD: Root mean squared deviation
ROC: Receiver operating characteristic curve
STRING: Search tool for retrieval of interacting genes
