# Supplementary figures and images for: Ten-gene signature reveals the significance of clinical prognosis and immuno-correlation of osteosarcoma and study on novel skeleton inhibitors regarding MMP9
Source: Cancer Cell Int. 2021 Jul 14;21:377. doi: 10.1186/s12935-021-02041-4 (PMC8281696; doi:10.1186/s12935-021-02041-4)

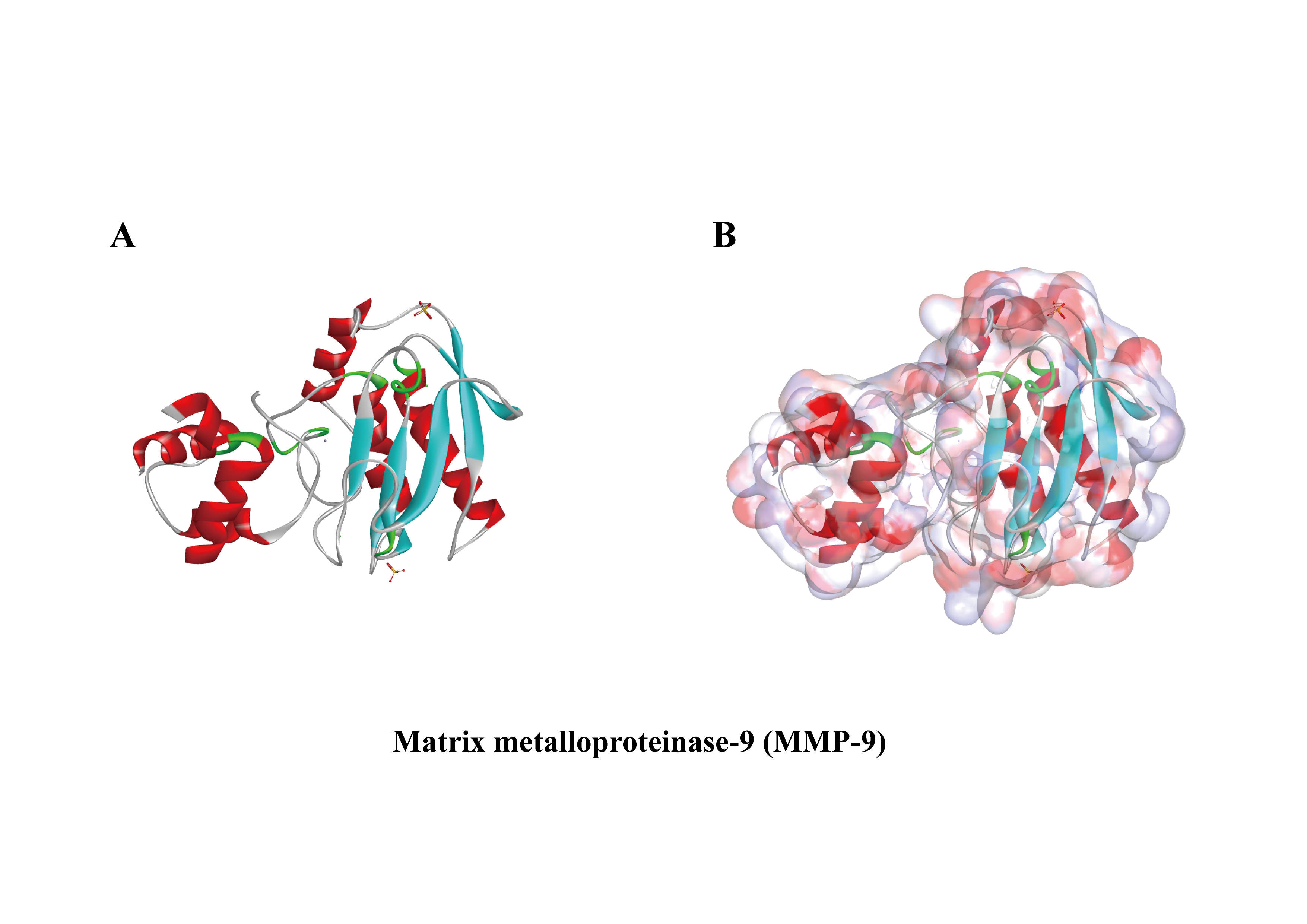

Supplement: Supplementary file 1 — Additional file 1: Figure S1. Crystal structure of Matrix metalloproteinase-9 (MMP-9). (A), Initial crystal structure. (B), Bing surface added. Blue represented positive charge and red represented negative charge. [file 12935_2021_2041_MOESM1_ESM.tif]

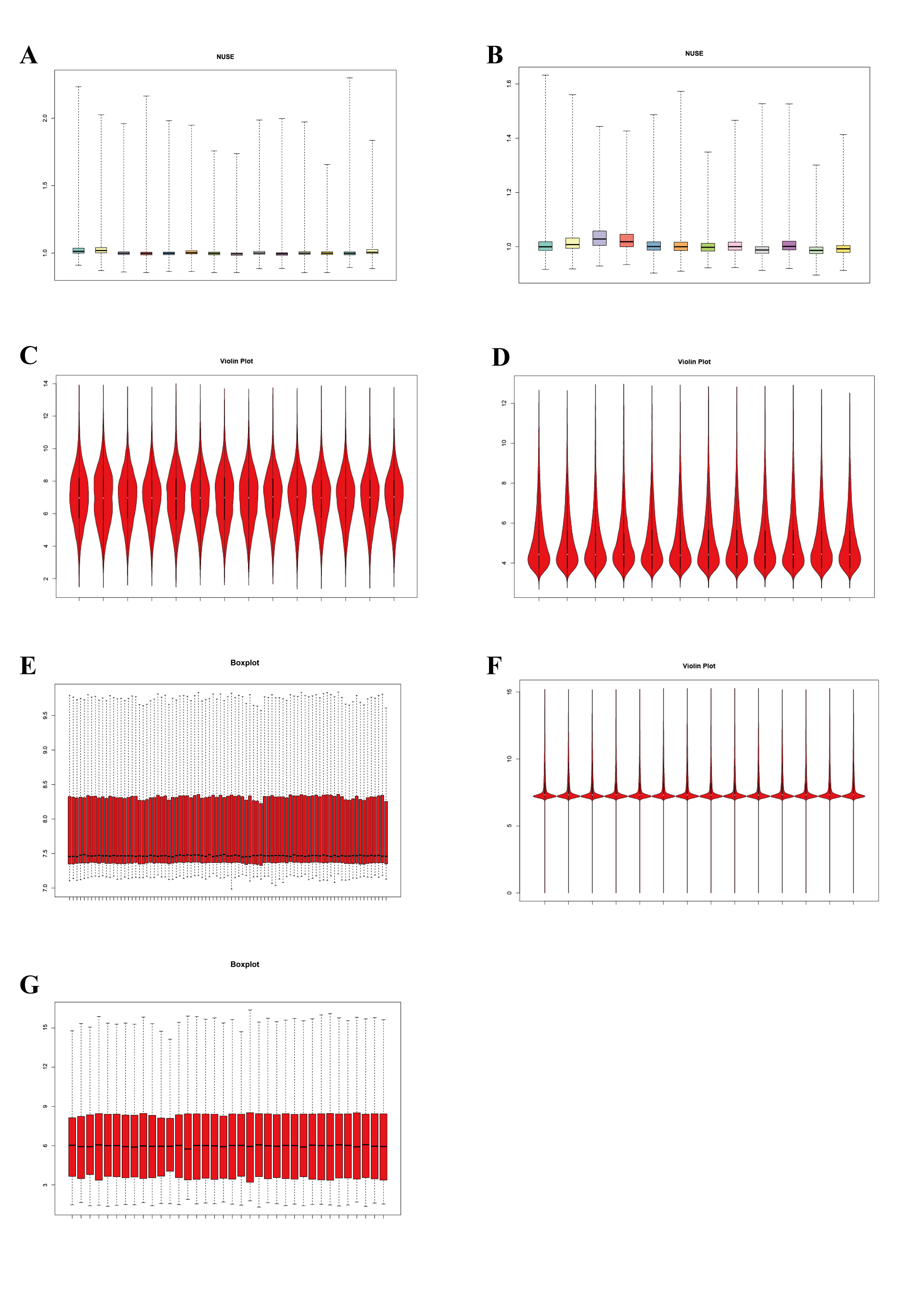

Supplement: Supplementary file 2 — Additional file 2: Figure S2. Boxplot of normalized unscaled standard errors (NUSE) of (A) GSE12865 and (B) GSE14359, which were used for quality control. Boxplot or violin plot after matrix background correction and normalization of (C) GSE12865; (D) GSE14359; (E) GSE33382; (F) GSE36001 and (G) GSE99671, which were displayed to visualize and verify the expression distribution. [file 12935_2021_2041_MOESM2_ESM.tif]

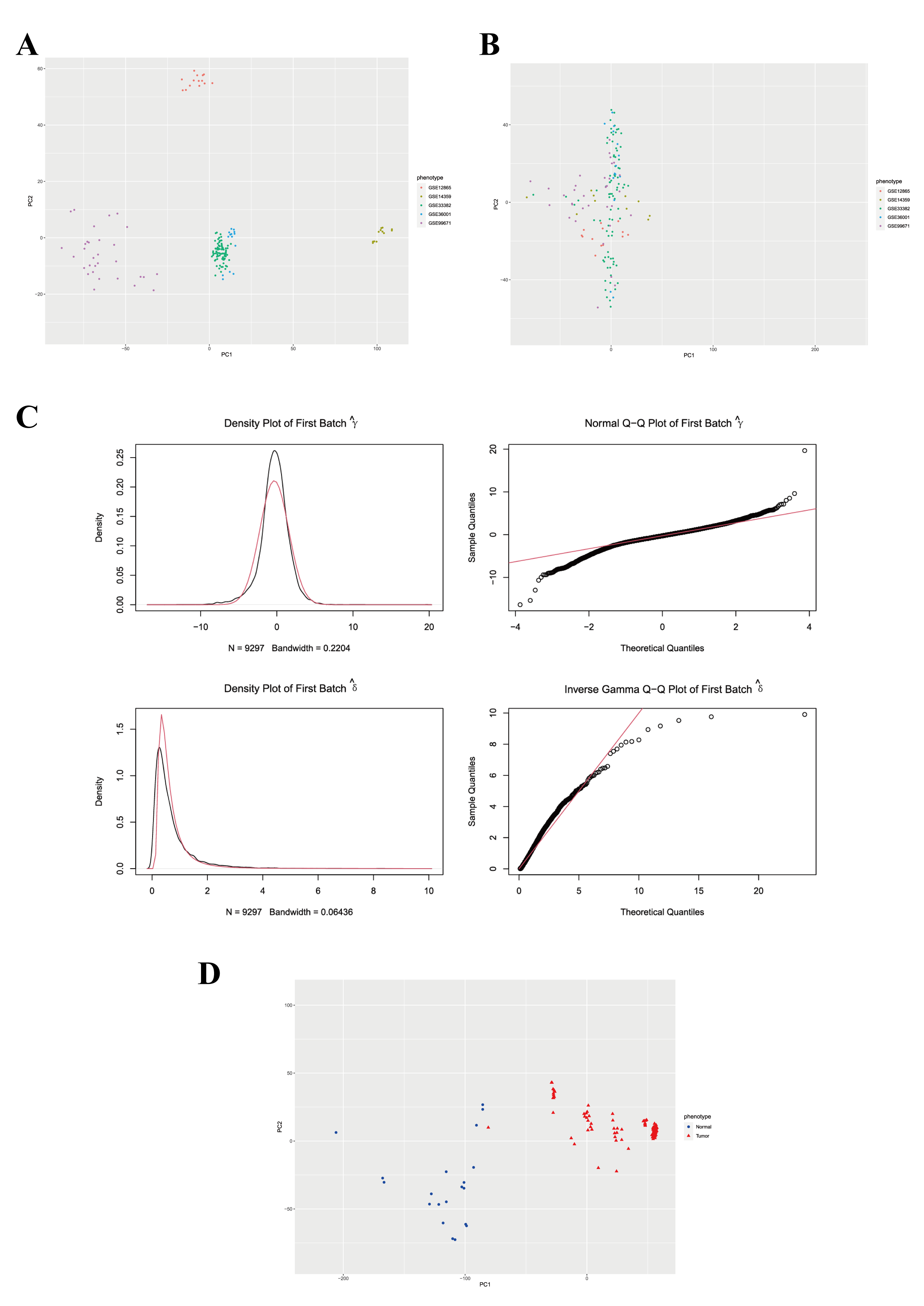

Supplement: Supplementary file 3 — Additional file 3: Figure S3. (A), PCA scatter plot among 5 series before removing “batch effects”. PCA: principal component analysis. (B), PCA scatter plot among 5 series after eliminating “batch effects”. (C), Generated QQ-plot and density plot after eliminating “batch effects”. (D), PCA analysis between different phenotypes based on the processed matrix, normal tissues could be distinguished from tumor tissues on PCA1 axis. [file 12935_2021_2041_MOESM3_ESM.tif]

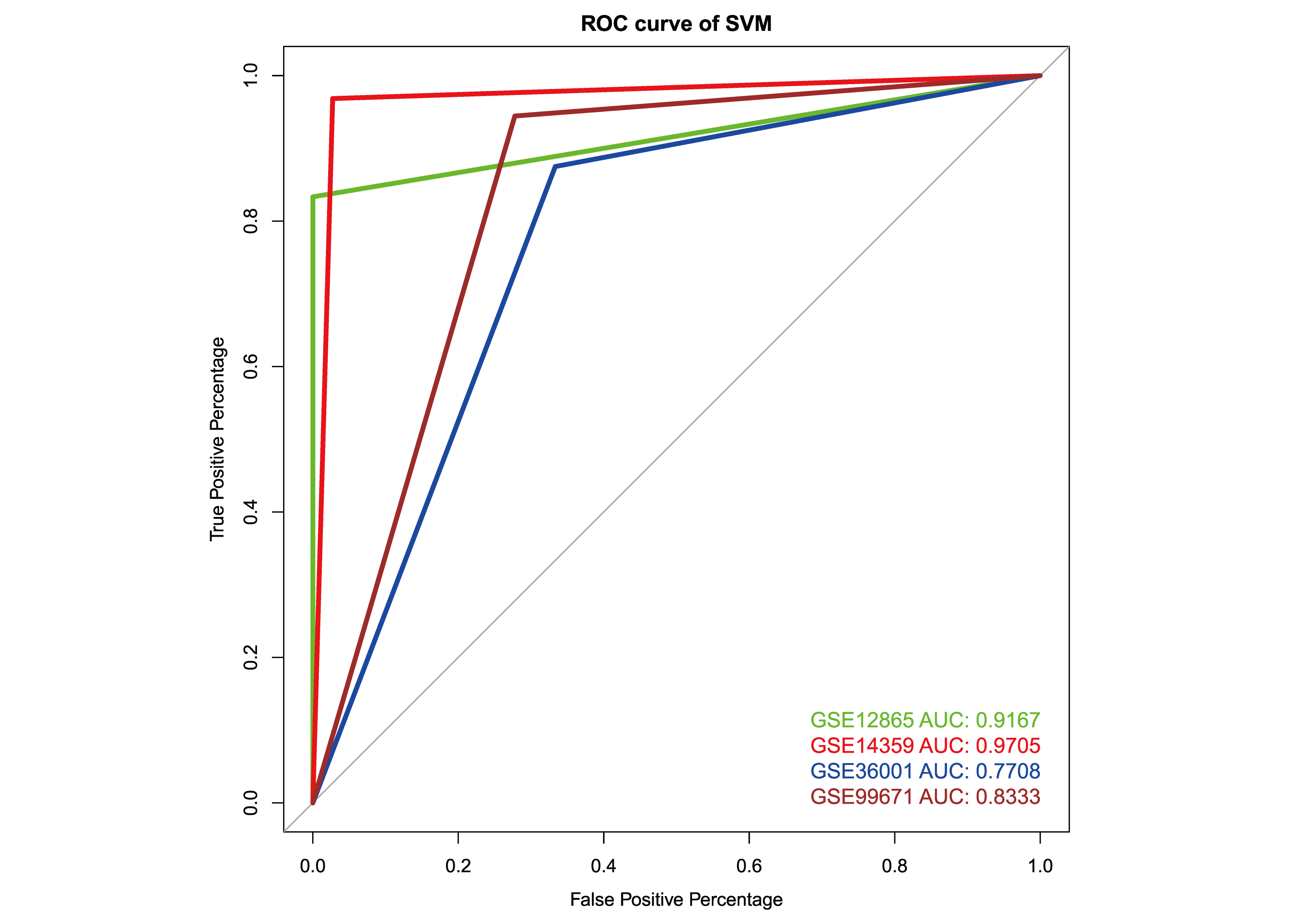

Supplement: Supplementary file 5 — Additional file 5: Figure S4. Area under curve of ROC diagram of support vector machine (SVM). [file 12935_2021_2041_MOESM5_ESM.tif]

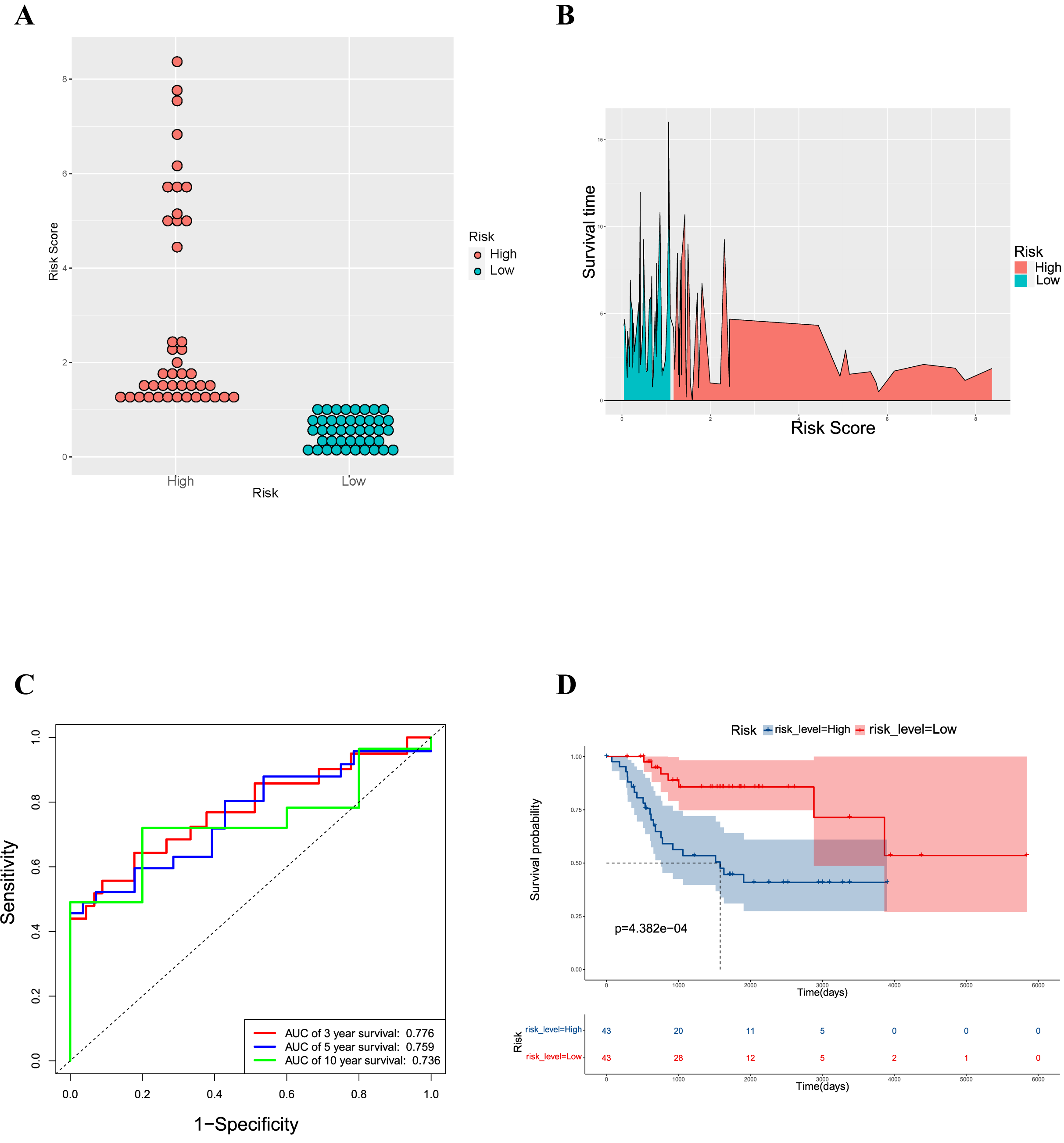

Supplement: Supplementary file 6 — Additional file 6: Figure S5. Construction of risk group base on RiskScore by multivariate Cox analysis. (A), Scatter plot of risk scores distribution in high-risk and low-risk group. (B), Overall survival time with different risk scores. (C), ROC curve of 10-gene signature for prognostic classification. (D), K-M survival prognosis of 10-gene signature model. [file 12935_2021_2041_MOESM6_ESM.tif]

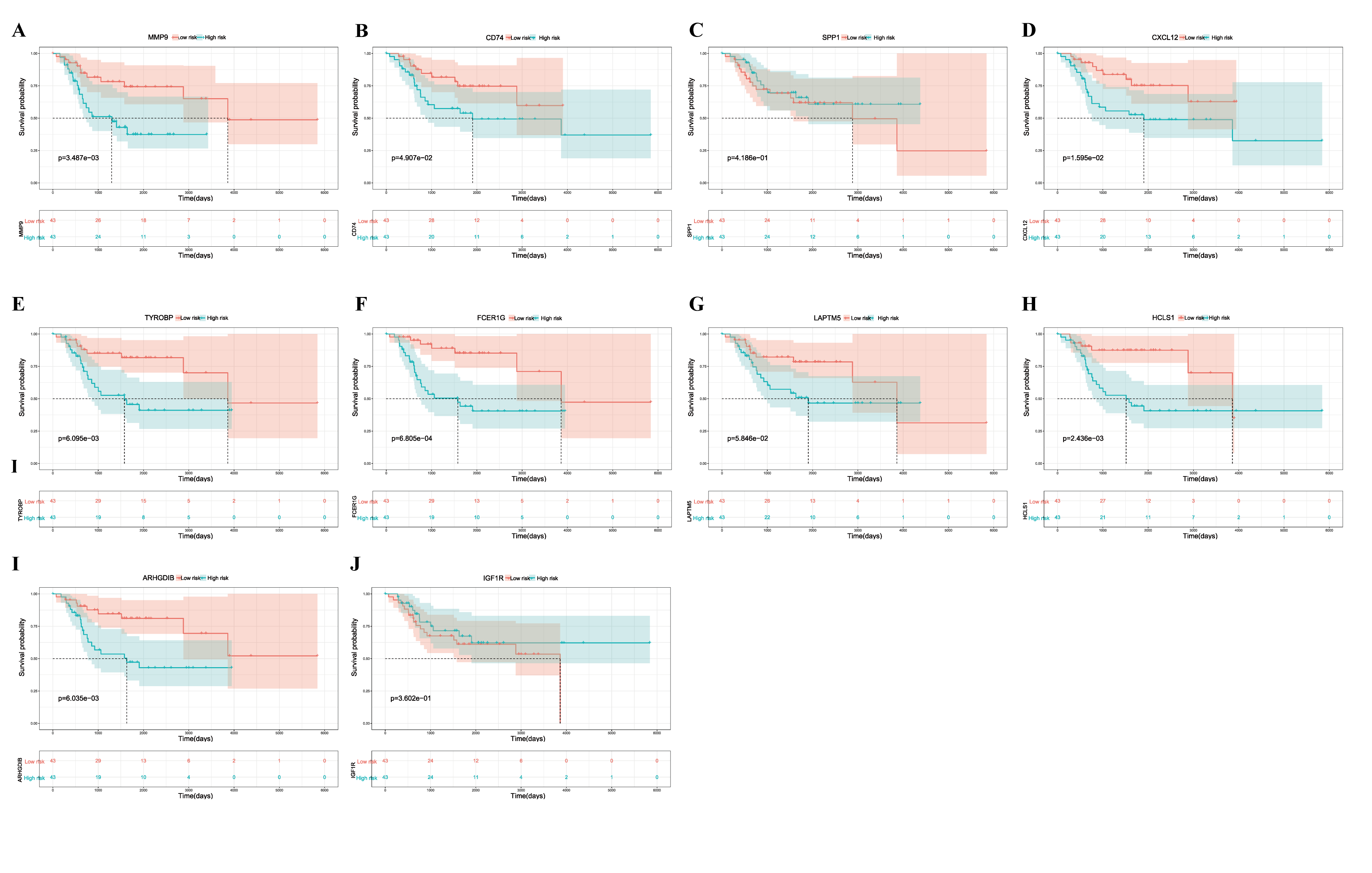

Supplement: Supplementary file 7 — Additional file 7: Figure S6. Kaplan–Meier overall survival analysis of 10 hub genes in osteosarcoma patients from TCGA database. (A), Survival analysis of MMP9 in osteosarcoma. (B), Survival analysis of CD74 in osteosarcoma. (C), Survival analysis of SPP1 in osteosarcoma. (D), Survival analysis of CXCL12 in osteosarcoma. (E), Survival analysis of TYROBP in osteosarcoma. (F), Survival analysis of FCER1G in osteosarcoma. (G), Survival analysis of LAPTM5 in osteosarcoma. (H), Survival analysis of HCLS1 in osteosarcoma. (I), Survival analysis of ARHGDIB in osteosarcoma. (J), Survival analysis of IGF1R in osteosarcoma. [file 12935_2021_2041_MOESM7_ESM.tif]

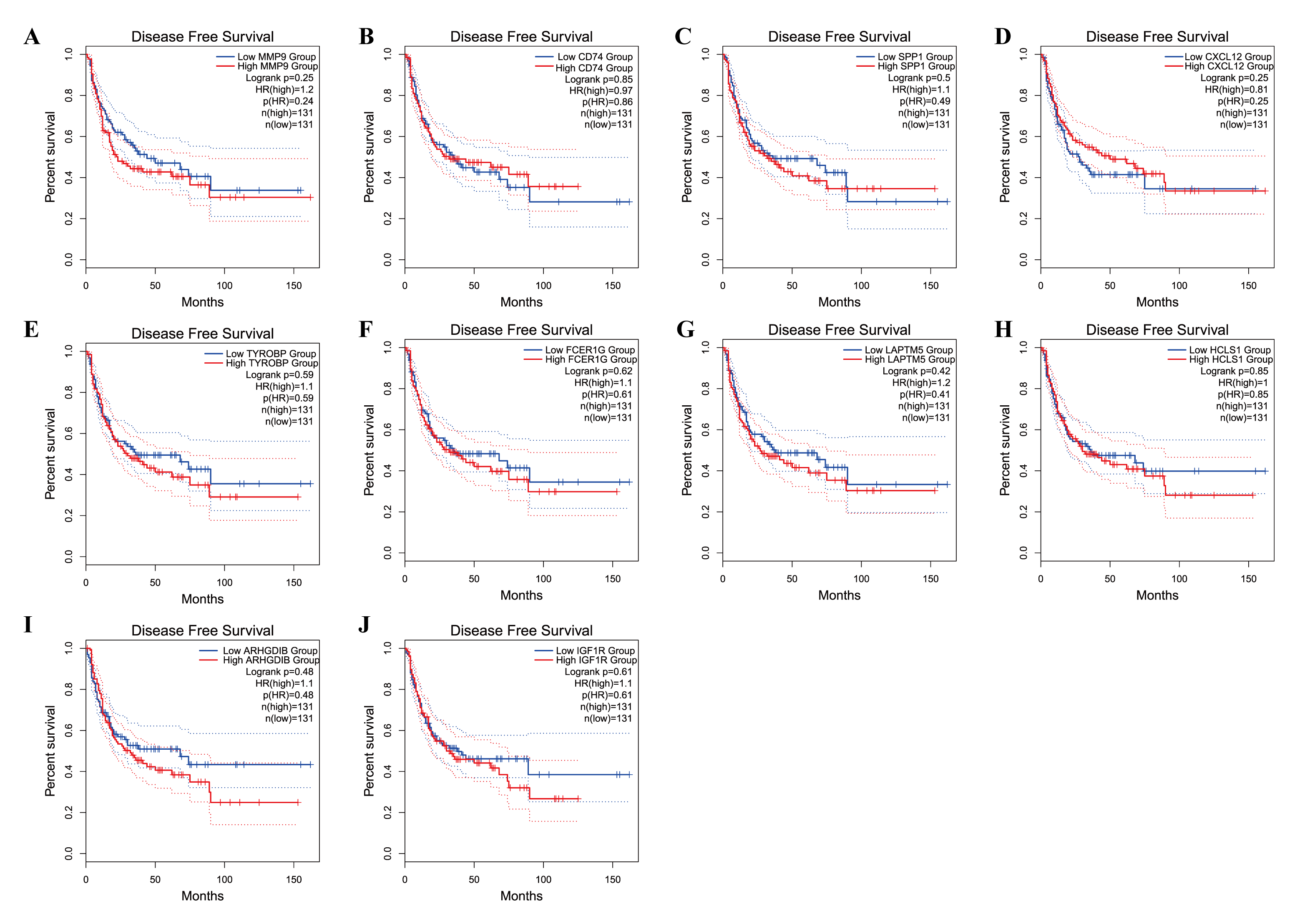

Supplement: Supplementary file 8 — Additional file 8: Figure S7. Disease-free survival (DFS) analysis of 10 hub genes in osteosarcoma patients from the GEPIA2 database. (A), Survival analysis of MMP9 in osteosarcoma. (B), Survival analysis of CD74 in osteosarcoma. (C), Survival analysis of SPP1 in osteosarcoma. (D), Survival analysis of CXCL12 in osteosarcoma. (E), Survival analysis of TYROBP in osteosarcoma. (F), Survival analysis of FCER1G in osteosarcoma. (G), Survival analysis of LAPTM5 in osteosarcoma. (H), Survival analysis of HCLS1 in osteosarcoma. (I), Survival analysis of ARHGDIB in osteosarcoma. (J), Survival analysis of IGF1R in osteosarcoma. [file 12935_2021_2041_MOESM8_ESM.tif]

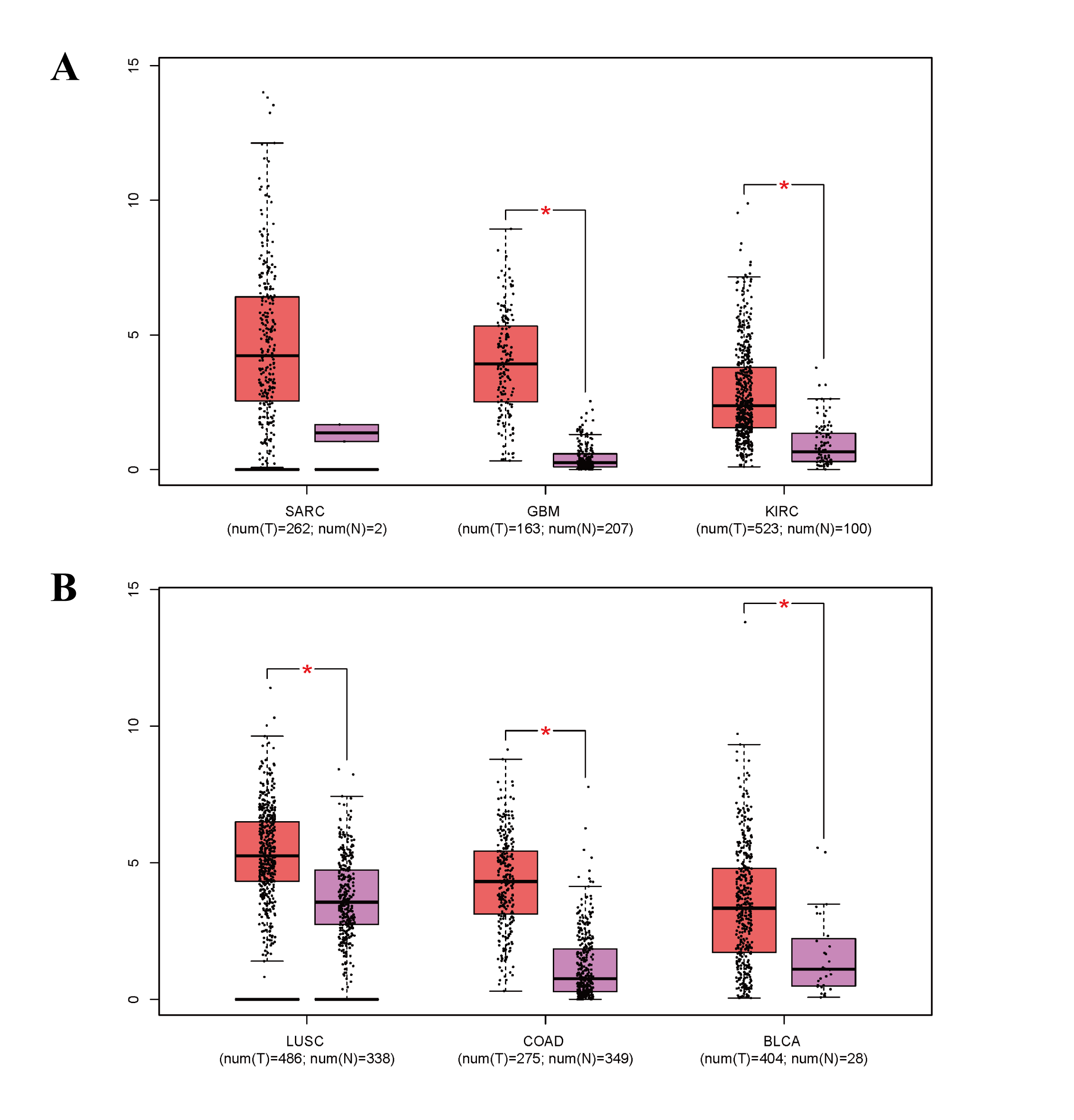

Supplement: Supplementary file 9 — Additional file 9: Figure S8. MMP9 expression patterns in different cancer types including SARC, GBM, KIRC, LUSC, COAD, BLCA. SARC: sarcoma; GBM: glioblastoma multiforme; KIRC: kidney renal clear cell carcinoma; LUSC: lung squamous cell carcinoma; COAD: colon adenocarcinoma; BLCA: bladder urothelial carcinoma. *P < 0.01. [file 12935_2021_2041_MOESM9_ESM.tif]

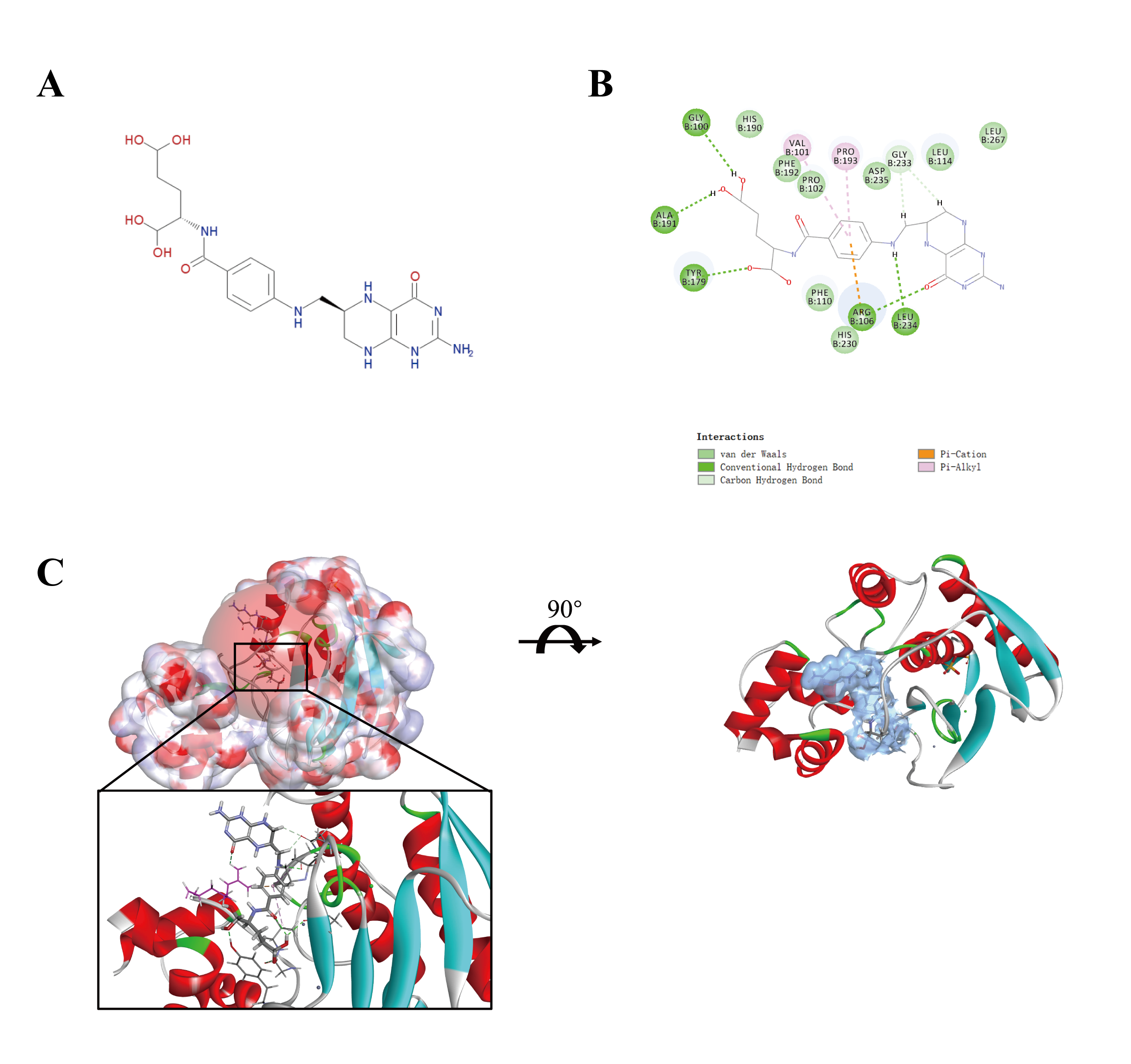

Supplement: Supplementary file 14 — Additional file 14: Figure S9. (A), Chemical structure of novel compound ZINC000004228235 selected from virtual screening. (B), Schematic drawing of inter-molecular interactions of the computed binding modes of ZINC000004228235with MMP9 based on highly precise docking method. (C), Visualization of chemical interactions between the ligand and MMP9 after highly precise docking method (ZINC000004228235-MMP9 complex). The surface of binding area as well as the active binding region were added. Blue represented positive charge, red represented negative charge and active binding region was shown with red sphere. Inhibitors was displayed with sticks, with the structures around ligand-receptor junction shown in thinner sticks. [file 12935_2021_2041_MOESM14_ESM.tif]

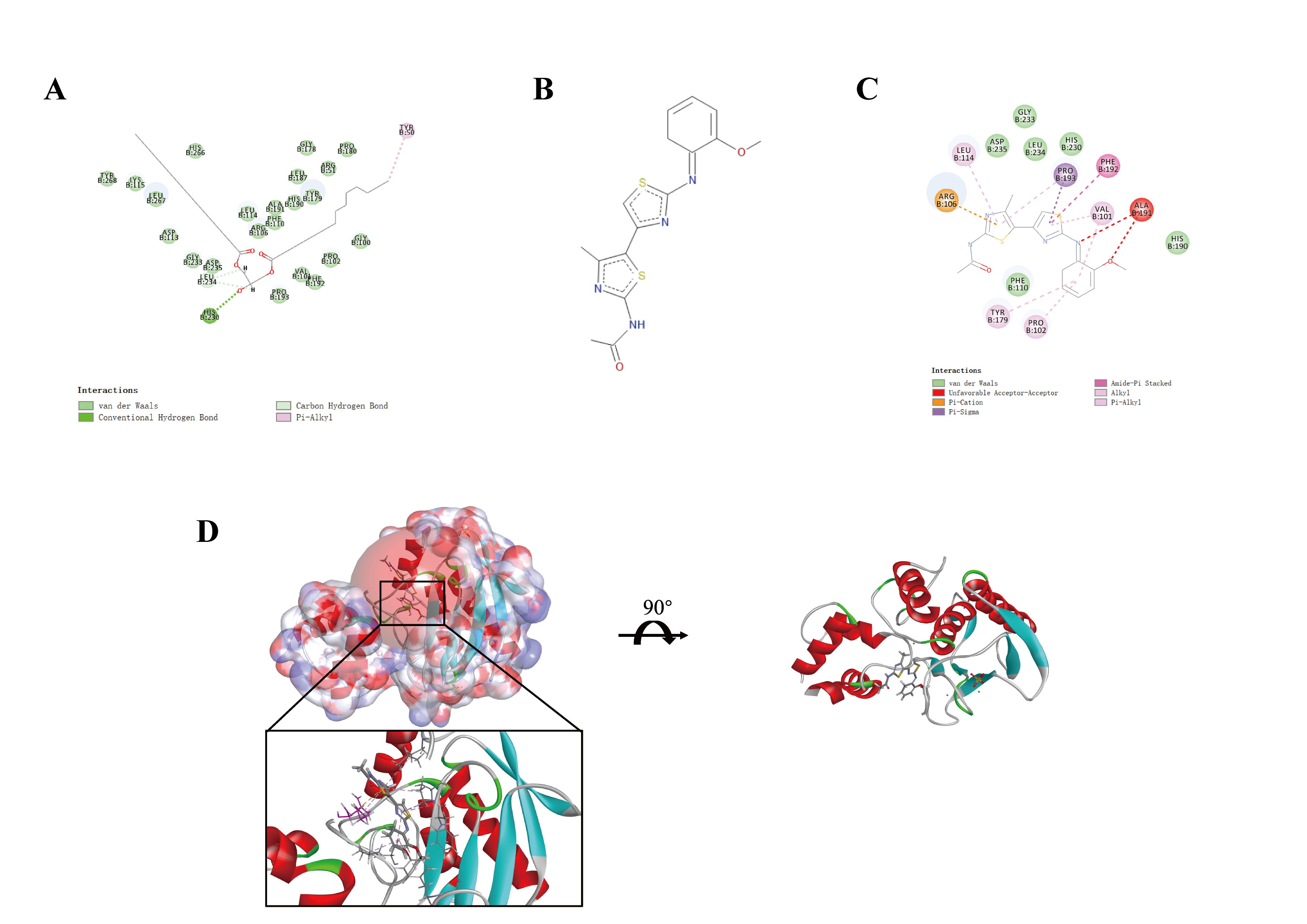

Supplement: Supplementary file 15 — Additional file 15: Figure S10. (A), Schematic drawing of inter-molecular interactions of the computed binding modes of ZINC000085810532 with MMP9 based on highly precise docking method. (B), Chemical structure of the reference ligand JNJ0966. (C), Schematic drawing of inter-molecular interactions of the computed binding modes of the reference ligand JNJ0966 with MMP9 based on highly precise docking method. (D), Visualization of chemical interactions between the reference ligand and MMP9 after highly precise docking method (JNJ0966-MMP9 complex). The surface of binding area as well as the active binding region were added. Blue represented positive charge, red represented negative charge and active binding region was shown with red sphere. Inhibitors was displayed with sticks, with the structures around ligand-receptor junction shown in thinner sticks. [file 12935_2021_2041_MOESM15_ESM.tif]
