# Supplementary material for: Ten-gene signature reveals the significance of clinical prognosis and immuno-correlation of osteosarcoma and study on novel skeleton inhibitors regarding MMP9
Source: Cancer Cell Int. 2021 Jul 14;21:377. doi: 10.1186/s12935-021-02041-4 (PMC8281696; doi:10.1186/s12935-021-02041-4)
Supplement: Supplementary file 4 — Additional file 4: Table S1. Detailed score information of hub genes through MCC algorithm. [file 12935_2021_2041_MOESM4_ESM.docx]

**Table S1**. Detailed score information of hub genes through MCC algorithm.

| Gene Symbol | Betweenness | MCC | Gene Symbol | Betweenness | MCC |
| --- | --- | --- | --- | --- | --- |
| MMP9 | 206.9869 | 7794 | GAS6 | 1.06349 | 1560 |
| CD74 | 74.46084 | 7458 | LTBP2 | 27.03596 | 1470 |
| SPP1 | 232.3187 | 7057 | A2M | 19.97647 | 312 |
| CXCL12 | 77.80932 | 6624 | RNASE1 | 8.57133 | 288 |
| TYROBP | 41.94157 | 6078 | IL3RA | 135.2794 | 56 |
| FCER1G | 20.21133 | 6024 | CYP1B1 | 97.18952 | 50 |
| LAPTM5 | 14.09951 | 5766 | PTX3 | 0.22222 | 48 |
| HCLS1 | 14.6992 | 5766 | COL10A1 | 31.0327 | 25 |
| ARHGDIB | 10.83466 | 5766 | TM4SF1 | 68.61905 | 9 |
| IGF1R | 46.07507 | 5076 | RB1 | 0 | 6 |
| GIMAP4 | 1.7 | 5064 | ADH1B | 4.83333 | 3 |
| HLA-DPA1 | 0 | 5040 | IL9R | 68 | 2 |
| SERPINE1 | 130.3598 | 3879 | CRLF2 | 0 | 2 |
| TAGLN | 1.74921 | 3600 | CPE | 0 | 2 |
| MMP13 | 9.21333 | 2928 | AKR1C3 | 0 | 2 |
| ALPL | 12.31467 | 2928 | PNMA2 | 0 | 1 |
| ANPEP | 76.41631 | 2216 | LRRC17 | 0 | 1 |
| IGFBP4 | 14.98612 | 1566 | DDAH1 | 0 | 1 |
