# Supplementary material for: Ten-gene signature reveals the significance of clinical prognosis and immuno-correlation of osteosarcoma and study on novel skeleton inhibitors regarding MMP9
Source: Cancer Cell Int. 2021 Jul 14;21:377. doi: 10.1186/s12935-021-02041-4 (PMC8281696; doi:10.1186/s12935-021-02041-4)
Supplement: Supplementary file 10 — Additional file 10: Table S2. The top 20 ranked compounds with higher libdock scores as well as the reference compound JNJ0966. [file 12935_2021_2041_MOESM10_ESM.docx]

**Table S2.** The top 20 ranked compounds with higher libdock scores as well as the reference compound JNJ0966.

| Number | Compounds | Libdock score |
| --- | --- | --- |
| 1 | ZINC000095620524 | 223.934 |
| 2 | ZINC000008552069 | 220.448 |
| 3 | ZINC000062238222 | 215.678 |
| 4 | ZINC000004654845 | 203.273 |
| 5 | ZINC000085545908 | 202.317 |
| 6 | ZINC000085544839 | 200.874 |
| 7 | ZINC000085826837 | 200.585 |
| 8 | ZINC000004096684 | 200.389 |
| 9 | ZINC000004099068 | 198.04 |
| 10 | ZINC000085541163 | 196.7 |
| 11 | ZINC000072131515 | 195.256 |
| 12 | ZINC000004096653 | 194.715 |
| 13 | ZINC000085810532 | 193.988 |
| 14 | ZINC000004228235 | 193.323 |
| 15 | ZINC000085826835 | 193.061 |
| 16 | ZINC000073220104 | 192.238 |
| 17 | ZINC000013513540 | 192.167 |
| 18 | ZINC000003830635 | 191.335 |
| 19 | ZINC000004096878 | 191.098 |
| 20 | ZINC000049878510 | 190.773 |
| 21 | JNJ0966 | 116.113 |
