# Supplementary material for: Ten-gene signature reveals the significance of clinical prognosis and immuno-correlation of osteosarcoma and study on novel skeleton inhibitors regarding MMP9
Source: Cancer Cell Int. 2021 Jul 14;21:377. doi: 10.1186/s12935-021-02041-4 (PMC8281696; doi:10.1186/s12935-021-02041-4)
Supplement: Supplementary file 11 — Additional file 11: Table S3. ADME (absorption, distribution, metabolism and excretion) properties of the top 20 compounds. [file 12935_2021_2041_MOESM11_ESM.docx]

**Table S3**. ADME (absorption, distribution, metabolism and excretion) properties of the top 20 compounds.

| Number | Compounds | Solubility Level ^1^ | BBB Level ^2^ | Absorption Level ^3^ | CYP2D6 ^4^ | Hepatotoxicity ^5^ | PPB level ^6^ |
| --- | --- | --- | --- | --- | --- | --- | --- |
| 1 | ZINC000095620524 | 4 | 4 | 3 | 0 | 1 | 0 |
| 2 | ZINC000008552069 | 4 | 4 | 3 | 0 | 1 | 0 |
| 3 | ZINC000062238222 | 3 | 4 | 3 | 0 | 1 | 0 |
| 4 | ZINC000004654845 | 1 | 4 | 3 | 1 | 0 | 1 |
| 5 | ZINC000085545908 | 4 | 4 | 3 | 0 | 0 | 0 |
| 6 | ZINC000085544839 | 3 | 4 | 3 | 0 | 1 | 0 |
| 7 | ZINC000085826837 | 2 | 4 | 2 | 0 | 0 | 0 |
| 8 | ZINC000004096684 | 1 | 4 | 3 | 0 | 0 | 1 |
| 9 | ZINC000004099068 | 3 | 4 | 3 | 0 | 0 | 0 |
| 10 | ZINC000085541163 | 2 | 4 | 2 | 0 | 0 | 0 |
| 11 | ZINC000072131515 | 3 | 4 | 3 | 0 | 0 | 1 |
| 12 | ZINC000004096653 | 1 | 4 | 3 | 0 | 0 | 1 |
| 13 | ZINC000085810532 | 2 | 4 | 3 | 0 | 0 | 1 |
| 14 | ZINC000004228235 | 3 | 4 | 3 | 0 | 0 | 0 |
| 15 | ZINC000085826835 | 2 | 4 | 2 | 0 | 0 | 0 |
| 16 | ZINC000073220104 | 1 | 4 | 3 | 0 | 0 | 1 |
| 17 | ZINC000013513540 | 4 | 4 | 3 | 0 | 1 | 0 |
| 18 | ZINC000003830635 | 5 | 4 | 3 | 0 | 0 | 0 |
| 19 | ZINC000004096878 | 1 | 4 | 3 | 0 | 1 | 1 |
| 20 | ZINC000049878510 | 1 | 4 | 3 | 0 | 0 | 0 |
| 21 | JNJ0966 (reference) | 2 | 4 | 2 | 0 | 1 | 0 |

^1^ Aqueous-solubility level: 0 (extremely low); 1 (very low, but possible); 2 (low); 3 (good);

^2^ Blood Brain Barrier level: 0 (Very high penetrant); 1 (High); 2 (Medium); 3 (Low); 4 (Undefined);

^3^ Human-intestinal absorption level: 0 (good); 1 (moderate); 2 (poor); 3 (very poor);

^4^ Cytochrome P450 2D6 inhibition: 0 (Non-inhibition); 1 (Inhibition);

^5^ Hepatotoxicity: 0 (Non-toxic); 1 (Toxic);

^6^ Plasma Protein Binding: 0 (Absorbent weakly); 1 (Absorbent strong)
