# Supplementary material for: Ten-gene signature reveals the significance of clinical prognosis and immuno-correlation of osteosarcoma and study on novel skeleton inhibitors regarding MMP9
Source: Cancer Cell Int. 2021 Jul 14;21:377. doi: 10.1186/s12935-021-02041-4 (PMC8281696; doi:10.1186/s12935-021-02041-4)
Supplement: Supplementary file 12 — Additional file 12: Table S4. Toxicity predictions of the top 20 compounds. [file 12935_2021_2041_MOESM12_ESM.docx]

**Table S4.** Toxicity predictions of the top 20 compounds.

| Number | Compounds | AMES | FDA: Mouse | | FDA: Rat | | Weight of evidence carcinogenicity |
| --- | --- | --- | --- | --- | --- | --- | --- |
|  |  |  | Male | Female | Male | Female |  |
| 1 | ZINC000095620524 | 0 | 0.998 | 1 | 1 | 0 | 1 |
| 2 | ZINC000008552069 | 1 | 0 | 0 | 0 | 0 | 0.150 |
| 3 | ZINC000062238222 | 0.989 | 0 | 0 | 0 | 0 | 0.132 |
| 4 | ZINC000004654845 | 1 | 0.001 | 0 | 0 | 0.119 | 0 |
| 5 | ZINC000085545908 | 0 | 0 | 0 | 0 | 0 | 0 |
| 6 | ZINC000085544839 | 0.999 | 0 | 0 | 0 | 0 | 0.057 |
| 7 | ZINC000085826837 | 0 | 0.001 | 1 | 0 | 0.152 | 0.362 |
| 8 | ZINC000004096684 | 1 | 0 | 0 | 0 | 0.018 | 0 |
| 9 | ZINC000004099068 | 0.002 | 0 | 0 | 0 | 0.034 | 0 |
| 10 | ZINC000085541163 | 0 | 0.001 | 1 | 0 | 0.152 | 0.362 |
| 11 | ZINC000072131515 | 1 | 1 | 0 | 0 | 0.003 | 0 |
| 12 | ZINC000004096653 | 1 | 0 | 0 | 0 | 0.246 | 0 |
| 13 | ZINC000085810532 | 0 | 0.730 | 0.987 | 1 | 1 | 1 |
| 14 | ZINC000004228235 | 0.091 | 0.998 | 0.002 | 0 | 0 | 0.104 |
| 15 | ZINC000085826835 | 0 | 0.001 | 1 | 0 | 0.152 | 0.362 |
| 16 | ZINC000073220104 | 0 | 0.897 | 1 | 1 | 1 | 1 |
| 17 | ZINC000013513540 | 0 | 0.981 | 1 | 0.994 | 1 | 0.248 |
| 18 | ZINC000003830635 | 0.998 | 0.984 | 0.027 | 0.984 | 1 | 0.670 |
| 19 | ZINC000004096878 | 1 | 0.002 | 0 | 0 | 0.993 | 0.806 |
| 20 | ZINC000049878510 | 0 | 0.931 | 1 | 1 | 1 | 0 |
| 21 | JNJ0966 (reference) | 1 | 0.160 | 1 | 1 | 1 | 1 |
