# Supplementary material for: Ten-gene signature reveals the significance of clinical prognosis and immuno-correlation of osteosarcoma and study on novel skeleton inhibitors regarding MMP9
Source: Cancer Cell Int. 2021 Jul 14;21:377. doi: 10.1186/s12935-021-02041-4 (PMC8281696; doi:10.1186/s12935-021-02041-4)
Supplement: Supplementary file 13 — Additional file 13: Table S5. CDOCKER interaction energy of compounds with Matrix metalloproteinase-9 (MMP-9). [file 12935_2021_2041_MOESM13_ESM.docx]

**Table S5.** CDOCKER interaction energy of compounds with Matrix metalloproteinase-9 (MMP-9).

| Complex | CDOCKER interaction energy (Kcal/mol) |
| --- | --- |
| **ZINC000072131515-MMP9** | -55.6816 Kcal/mol |
| **ZINC000004228235-MMP9** | -56.348 Kcal/mol |
| **ZINC000085810532-MMP9** | -62.1737 Kcal/mol |
| **JNJ0966-MMP9** | -37.6049 Kcal/mol |
