# Supplementary material for: Ten-gene signature reveals the significance of clinical prognosis and immuno-correlation of osteosarcoma and study on novel skeleton inhibitors regarding MMP9
Source: Cancer Cell Int. 2021 Jul 14;21:377. doi: 10.1186/s12935-021-02041-4 (PMC8281696; doi:10.1186/s12935-021-02041-4)
Supplement: Supplementary file 16 — Additional file 16: Table S6. Chemical bond interaction parameters of each compound with MMP9 residues. [file 12935_2021_2041_MOESM16_ESM.docx]

**Table S6.** Chemical bond interaction parameters of each compound with MMP9 residues.

| Receptor | Compound | Interaction residues | Distances (Å) | Chemical bond type |
| --- | --- | --- | --- | --- |
| MMP9 | ZINC515 | ZINC515:O35--PRO180: HD1 | 2.54 | Carbon hydrogen bond |
|  |  | ZINC515--ARG51: NH1 | 3.52 | Pi-Cation bond |
|  |  | ZINC515--TYR179 | 5.14 | Pi-Pi interaction bond |
|  |  | ZINC515:C8--LEU267 | 4.89 | Alkyl bond |
|  |  | ZINC515:C18--VAL101 | 4.56 | Alkyl bond |
|  |  | ZINC515:C23--PRO102 | 4.72 | Alkyl bond |
|  |  | ZINC515:C23--ARG106 | 4.94 | Alkyl bond |
|  |  | ZINC515:C28--PRO102 | 3.55 | Alkyl bond |
|  |  | ZINC515:C33--LEU187 | 4.76 | Alkyl bond |
|  |  | ZINC515:C23--PHE110 | 4.79 | Pi-Alkyl bond |
|  |  | ZINC515:C28--TYR179 | 4.03 | Pi-Alkyl bond |
|  |  | ZINC515:C18--HIS230 | 4.62 | Pi-Alkyl bond |
|  |  | ZINC515:C8--HIS266 | 4.49 | Pi-Alkyl bond |
|  |  | ZINC515--ARG51 | 5.46 | Pi-Alkyl bond |
|  | ZINC235 | ZINC235: O5--ARG106: HH21 | 2.25 | Hydrogen bond |
|  |  | ZINC235: O28--TYR179: HH | 1.90 | Hydrogen bond |
|  |  | ZINC235: H41--LEU234: O | 2.51 | Hydrogen bond |
|  |  | ZINC235: H51--GLY100: O | 2.80 | Hydrogen bond |
|  |  | ZINC235: H51--ALA191: O | 2.44 | Hydrogen bond |
|  |  | ZINC235: H52-- ZINC235: O18 | 2.35 | Hydrogen bond |
|  |  | ZINC235: H36--GLY233: O | 2.46 | Carbon hydrogen bond |
|  |  | ZINC235: H40--GLY233: O | 2.60 | Carbon hydrogen bond |
|  |  | ZINC235--ARG106: NH1 | 3.77 | Pi-Cation bond |
|  |  | ZINC235--VAL101 | 4.81 | Pi-Alkyl bond |
|  |  | ZINC235--PRO193 | 4.10 | Pi-Alkyl bond |
|  | ZINC532 | ZINC532: O19--HIS230: HD1 | 2.77 | Hydrogen bond |
|  |  | ZINC532: H65--LEU234: O | 2.41 | Carbon hydrogen bond |
|  |  | ZINC532: H66--LEU234: O | 2.93 | Carbon hydrogen bond |
|  |  | ZINC532: C36--TYR50 | 5.10 | Pi-Alkyl bond |
|  | JNJ0966 (reference) | 5UE4--ARG106: NE | 4.41 | Pi-Cation bond |
|  |  | --PRO193: HD2 | 2.56 | Pi-Sigma bond |
|  |  | --PHE192:C, O; PRO193: N | 4.90 | Amide-Pi Stacked |
|  |  | --VAL101 | 4.90 | Alkyl bond |
|  |  | --PRO102 | 4.22 | Alkyl bond |
|  |  | --TYR179 | 5.16 | Pi-Alkyl bond |
|  |  | --VAL101 | 4.37 | Pi-Alkyl bond |
|  |  | --ARG106 | 5.45 | Pi-Alkyl bond |
|  |  | --LEU114 | 5.22 | Pi-Alkyl bond |
|  |  | --PRO193 | 5.05 | Pi-Alkyl bond |

ZINC515: **ZINC000072131515; ZINC235: ZINC000004228235; ZINC532: ZINC000085810532.**
